# Preparation, Digestion, and Storage of Microencapsulated Nervonic Acid-Enriched Structured Phosphatidylcholine

**DOI:** 10.3390/molecules30092007

**Published:** 2025-04-30

**Authors:** Xun Ang, Hong Chen, Jiqian Xiang, Fang Wei, Siew Young Quek

**Affiliations:** 1 Food Science Programme, School of Chemical Sciences, The University of Auckland, Auckland 1142, New Zealand; xang920@aucklanduni.ac.nz; 2 Riddet Institute, Centre for Research Excellence, Palmerston North 4474, New Zealand; 3 The Key Lab for Biological Sciences of Oil Crops, Ministry of Agriculture-Hubei Key Laboratory of Lipid Chemistry and Nutrition, Institute of Oil Crops Research, Chinese Academy of Agricultural Sciences, Wuhan 430062, China; chenhong@oilcrops.cn (H.C.); willasa@163.com (F.W.); 4 Enshi Autonomous Prefecture Academy of Agricultural Sciences, Enshi 445002, China; hmxjq@163.com

**Keywords:** nervonic acid, encapsulation, storage, digestion, conventional emulsion, microcapsule

## Abstract

This study focuses on the encapsulation of nervonic acid-enriched structured phospholipid (NA-enriched SPL) by analysing its physical and chemical properties. Wall materials for encapsulation were initially screened, with whey protein isolate and maltodextrin exhibiting the most favourable characteristics. Optimisation of encapsulation parameters determined that a core-to-wall ratio of 1:3 provided the highest physical stability. Encapsulated samples underwent in vitro digestion, where MC-FD exhibited the highest digestibility (79.54%), followed by CV-E (72.1%) and NA-enriched SPL (29.82%). Storage stability was assessed over 90 days at 4 °C, 25 °C, and 45 °C by monitoring particle size, zeta potential, polydispersity index, microscopy, fatty acid composition, and primary and secondary lipid oxidation. MC-FD demonstrated superior stability, maintaining its physical and chemical properties, particularly at 4 °C. In contrast, CV-E showed the lowest physical stability, with significant changes in appearance and increased particle size at elevated temperatures (25 °C and 45 °C).

## 1. Introduction

Nervonic acid (NA), a very long-chain monounsaturated fatty acid (C24:1, ω-9), is integral to the health and function of the central nervous system. It plays a key role in maintaining the health of nerve cells by supporting the production of myelin, the protective sheath surrounding neurons [1]. This myelin is essential for the efficient transmission of nerve signals. Deficiencies in myelin are linked to several neurodegenerative diseases, including multiple sclerosis (MS) and Alzheimer’s disease [1,2,3]. However, NA in free fatty acid (FFA) form is known to have poor absorption; hence, research by Ang et al. [4] prepared a NA-enriched structured phosphatidylcholine (NA-enriched SPC) to potentially improve the bioavailability of NA.

Encapsulation is a technique applied to entrap sensitive bioactive ingredients within a coating or wall material [5]. The wall material helps to improve the thermal stability, oxidative stability, and shelf-life of the bioactive ingredients [6]. This technology enables the controlled release of the bioactive compound to maximise its absorption in the body. Recently, the ultrasonic hot homogenisation method has been utilised in studies to encapsulate oils to obtain an emulsion, as studies have demonstrated that this method decreases emulsion droplet size, increasing the entrapment efficiency (EE) and stability [7]. Freeze drying is preferred as it prevents thermal degradation during the drying process [8], making it suitable for the preservation of NA.

The wall materials used to encapsulate bioactive compounds should have high solubility; good emulsification, film-forming, and drying properties; and provide an emulsion with low viscosity and protection against oxidation [9]. This is because the wall materials greatly influence the final product’s physical and functional properties [10]. Food-grade wall materials such as proteins, celluloses, gums, and starches are commonly used in the food industry depending on the core material [11]. Maltodextrin (MD), gum Arabic (GA), and whey protein isolate (WPI) are the most frequently used wall materials for the encapsulation of oils [12]. The choice of wall material is also an important criterion in the optimisation of encapsulation as it affects both the emulsion properties (viscosity, stability, and droplet size) and particle characteristics (surface oil, particle size, morphology, and oxidative stability during drying and storage [13]. Hence, the wall materials, including MD, WPI, GA, and OSA starch, were screened and selected based on their optimal physical properties.

The digestibility of the CV-E and MC-FD containing NA-enriched SPC are crucial to evaluate its potential for food application. Studies have shown that the bioaccessibility of lipids depends on the gastrointestinal fate of the lipid phase surrounding them [14]. The co-ingestion of lipids capable of forming mixed micelles has been reported to be critical for its absorption [15]. However, the digestion and absorption of lipids are complex processes involving numerous biochemical and physiochemical events throughout the gastrointestinal tract [16]. Hence, in vitro digestion of the compounds may assist in improving and understanding the changes undergone during digestion in the gastrointestinal tract. Simulated oral digestion was excluded in the in vitro digestion model as the samples used, including CV-E and MC-FD, have very short residence times in the oral cavity [17]. In addition, liquid food is not important in the simulation of an oral phase if the meal does not contain starch [18].

Storage stability is one of the most critical factors related to the physical and chemical stability of the CV-E and MC-FD. It is necessary to study the effect of storage conditions as they affect storage stability, including the shelf life attributed to lipid oxidation mechanisms [19]. Lipid oxidation is one of the most problematic deterioration processes occurring in the storage of lipid-based formulations, leading to the development of polymerisation reactions, rancidity, and off flavours. Lipid oxidation may also generate undesirable biologically active species that play a role in inflammatory and cardiovascular disease processes [20]. Lipids, mainly unsaturated fatty acids, are prone to lipid oxidation, especially when stored under inappropriate conditions such as exposure to oxygen, high relative humidity, and temperature. Furthermore, the oxidation of encapsulated lipid has been proposed to be mechanistically different from bulk oil as the water–oil interface has a pronounced effect on susceptibility to oxidation [21]. Hence, understanding the causes of physical and chemical instability and the appropriate storage conditions of encapsulated NA-enriched SPC is essential. In addition, the stability of encapsulated NA-enriched SPC has not been reported. Another study by Jin et al. [22] encapsulated NA in nanoemulsions using plant- and animal-based emulsifiers (soy and whey protein isolate) to explore their potential for functional food applications, providing a useful benchmark for current encapsulation approaches.

This research aims to encapsulate NA-enriched SPC within the selected wall materials, including proteins and polysaccharides, to produce CV-E and MC-FD. In addition, it aims to investigate the stability of the encapsulated NA-enriched SPC under various storage conditions by examining the particle size, zeta potential, peroxide value (PV), thiobarbituric reactive substances (TBARS), and fatty acid composition. The formulations were optimised according to their particle size, polydispersity index (PDI), zeta potential, and EE. The optimised formulation was characterised and digested with an in vitro digestion model.

## 2. Results and Discussion

### 2.1. Characterisation of Conventional Emulsion

NA-enriched SPC was produced in our laboratory before encapsulation. The wall materials (WPI, GA, MD, and OSA) were screened by analysing their particle size, PDI, zeta potential, and EE as they are essential indicators for emulsion stability [10,23,24,25]. WPI was selected as the primary emulsifier for the encapsulation of SPC due to its good solubility, ability to dissolve in a wide range of pH, and inherent emulsifying potential [26]. For the encapsulation of hydrophobic core materials, obtaining stable emulsions before drying is crucial as it ensures the production of good-quality powders.

The most suitable combination of wall materials was selected by evaluating the particle size, PDI, zeta potential, and EE, as shown in Table 1. Even though WPI + MD has the smallest particle size of 174.58 ± 5.72 nm, in terms of particle size, there was no significant difference (*p* > 0.05) between the particle size of all the wall materials. This suggests that sufficient emulsifier was present to confer interfacial stability to emulsions. WPI was the primary emulsifier during the emulsion production as all the other parameters were kept constant, including the amount of emulsifiers (except WPI only) and the lipid content. Therefore, the combination of wall materials, especially polysaccharides, does not significantly affect the particle size. However, each emulsifier’s properties can differ in adsorption rate, molecular weight, conformational adjustment, reduction of interfacial tension, and excess surface concentration [27].

A study by Koç et al. [13] reported that larger particles were obtained when WPI was used as wall material compared to MD, possibly due to its higher emulsion viscosity. The authors also mentioned that the smallest particle sizes were obtained when the wall materials ratio of MD:WPI was equal. Chandralekha et al. [28] and Ozdemir et al. [29] reported that small particle size is related to lower viscosity, which is why combining the wall materials could help with lowering the viscosity to decrease average particle size. In this study, there may be a sufficient amount of emulsifier, and the use of MD did not affect the emulsifying capability of the system. WPI+GA and WPI+OSA showed higher PDIs of 0.331 and 0.308, possibly caused by their lower solubility and higher viscosity compared to WPI+MD. WPI has a relatively low PDI of 0.259. By comparing the PDI among the wall materials, WPI+MD is shown to have the lowest PDI of 0.169 with the narrowest particle size distribution.

The different combinations of wall materials used for the emulsion displayed significant differences (*p* < 0.05) in negative zeta potential ranging from −21.28 to −35.15 mV (Table 1). Emulsions exhibiting zeta potential within the range of +30 to −30 mV usually tend to coagulate or flocculate, while emulsions with higher than +30 mV or lower than −30 mV tend to be electrostatically stable [30]. WPI and WPI+MD demonstrated high stability with a zeta potential of −35.15 mV and −32.18 mV, respectively, while WPI+GA and WPI+OSA had lower zeta potentials of −23.15 and −21.28 mV, respectively. There was also a significant difference (*p* < 0.05) between both groups, possibly due to the higher concentration of WPI only, and WPI+MD may have had a superior synergy that produced a stable emulsion from increased emulsification capability that leaves a low amount of uncovered oil droplets. It is also possible that using two emulsifiers may result in the competition of adsorption on the oil surface. In addition, the negatively charged surface groups offer electrostatic repulsion among the emulsion droplets that prevents them from aggregation and increases their stability in the emulsion [12].

The EE of all combinations of wall materials is above 99%, and there is no significant difference between them (*p* > 0.05). This indicates that the wall materials have no significant influence on EE. It is possible that SPC as a natural emulsifying agent [31] and WPI as an emulsifier boosted the encapsulation ability of the emulsion system, resulting in a high EE of over 99%. The literature has reported that the oil concentration and wall materials used are critical factors for the encapsulation process [10]. The results indicate that the wall materials were effective in encapsulating the core material consisting of oil and SPC.

By comparing the particle size distribution in Supplementary Figure S1A, it was observed that their distribution varied with different wall materials. WPI has a profoundly broad tailing peak, showing that WPI has a poorer emulsifying ability. WPI may have a high emulsifier concentration that causes it to aggregate in solution, leading to a wide particle size distribution [23]. A hypothesis put forward by Euston et al. [32] mentioned that aggregation takes place between adsorbed protein at the emulsion droplet surface and non-adsorbed heat-denatured protein. Similar studies have also reported that aggregation is more rapid at higher WPI concentrations [32,33]. Another explanation could be the slower adsorption rate of WPI on the droplet surface, making it difficult to quickly coat the newly formed droplets due to their high molecular weights and complex structures [34]. Hence, a higher adsorption rate helps to fully coat the newly formed interface as rapidly as possible to prevent them from re-coalescence before new droplets leave the emulsification area [23]. Three peaks were observed in the particle size distribution of WPI+OSA, while WPI+GA displayed a bimodal size distribution. WPI+GA and WPI+OSA show an extra peak in the 4000–5000 nm range, likely due to aggregation, which could have been due to the occurrence of aggregation or clustering in polysaccharide solutions [35]. The development of aggregation is related to factors such as the type of wall materials, concentration, pH, homogenisation process, and others [36].

The particle size distribution and PDI of the WPI+MD emulsion demonstrated that it has a predominant homogeneous droplet size. In addition, WPI:MD emulsions were slightly smaller in size with lower zeta potential that conferred its stability and resisted aggregation. Based on the above results, WPI:MD wall materials were selected for further research to select the optimum core:wall ratio.

The results presented in Table 2 show a significant effect of core:wall ratio on the particle size, zeta potential, and EE. The core:wall ratio of 1:1 has the largest average particle size of 923.63 nm and decreases significantly to 188.35 nm as the ratio goes up to 1:3. When the core:wall ratio goes up to 1:4, only a slight decrease in particle size to 170.58 nm is observed, in which no significant difference (*p* > 0.05) is observed. This shows that at a core:wall ratio of 1:1, coalescence and aggregation of particles led to large particle sizes. Therefore, for the droplets to be small, sufficient wall materials are required to fully encapsulate the core material to prevent aggregation [12]. An increase in the core:wall ratio can result in coalescence or flocculation as insufficient emulsifiers coat the oil droplets [37]. A study by San et al. [38] reported similar results in which a decreased wall-to-oil ratio led to an increase in droplet size. A low wall-to-oil ratio of 1:1 also presented oil droplets on the surface after 24 h, indicating that the oil was not fully encapsulated.

**Figure 1:**
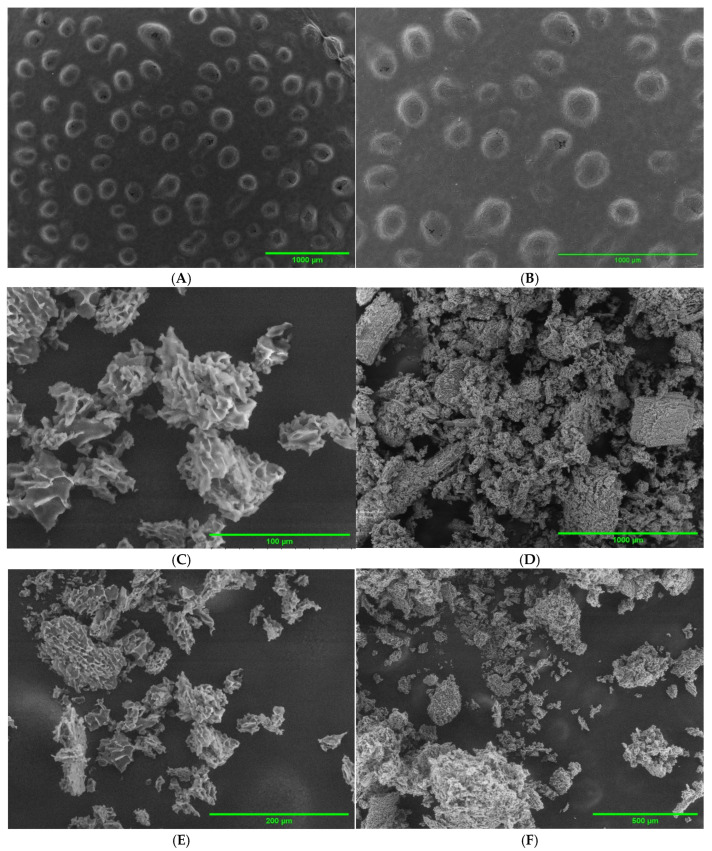
SEM images of optimised CV-E (**A**,**B**) and MC-FD (**C**–**F**).

The core:wall ratio of 1:1 displays a bimodal particle size distribution with a large peak at approximately 350–1000 nm and a smaller peak around 70–200 nm. It is likely caused by the agglomeration or coalescence of the smaller particles, as there were insufficient emulsifiers and wall materials to coat the droplets [37]. A core:wall ratio of 1:2 shows multimodal peaks with two conjoint peaks ranging from 70 nm to 1000 nm and a tiny peak at approximately 5000 nm. As the core:wall ratio increases to 1:3 and 1:4, the monomodal particle size distribution is shown in Figure 1B, which indicated that a narrow particle size distribution was obtained for both ratios. This indicates that the particle sizes were relatively similar and homogeneous. The particle size distribution results in Figure 1B agree with the PDI obtained in Table 2, in which PDI values below 0.3 or lower indicate uniformity in the particle sizes.

A negative correlation was observed between particle size, PDI, zeta potential, and EE as the core-to-wall ratio increased. This suggests that a higher core-to-wall ratio enhances formulation stability and homogeneity. A core:wall ratio of 1:2 displayed high emulsion stability with the highest zeta potential of −58.87 mV, followed by 1:1, 1:3, and 1:4. A significant decrease (*p* < 0.05) in zeta potential was observed as the core:wall ratio increased from 1:2 to 1:3. Generally, all the core:wall ratio formulations were more than ±30 mV, implicating that they can prevent emulsion droplets from aggregation and maintains good stability. Even though the core:wall ratio of 1:1 has a high zeta potential of −54.77 mV, it also has a very large average particle size. This could be due to the early aggregation of the particles during emulsification that may have already stabilised after aggregation, resulting in a high electrostatic repulsion. In addition, the insufficient wall materials resulted in high particle aggregation.

As the core:wall ratio increased from 1:1 to 1:4, a significant increase (*p* < 0.05) in EE from 95.01% to 99.47% was observed. This indicates that the increase in emulsifier (WPI+MD) amount led to an increase in EE. Tolve et al. [39] mention that the core:wall ratio strongly influences EE. Other studies have also reported that particle size influences EE, with larger sizes resulting in poorer EE [40]. This phenomenon can be observed in Table 2, where the EE decreases significantly (*p* < 0.05) as the core:wall ratio decreases from 1:3 to 1:2.

Based on the results shown in Table 2 and Figure 1B, a core:wall ratio of 1:3 was selected as the optimum ratio before lyophilisation. The reason was the inferior particle size, PDI, and EE when the core:wall ratio was 1:1 and 1:2 as compared to core:wall ratios of 1:3 and 1:4, except for their zeta potential, which displayed better stability. However, ultimately, the core:wall ratio of 1:3 was still chosen as there were no significant differences between the core:wall ratios of 1:3 and 1:4. Furthermore, increasing the loading of the core material without affecting its stability is beneficial. Some researchers have also reported that a higher wall:core ratio could interfere with lipase activity and reduce the digestibility of lipids [41].

### 2.2. SEM Imaging

CV-E, as shown in Figure 1A,B, demonstrated a spherical shape with fissures and cracking on the surface. Based on the scale bar, the particle size is approximately 200 µm. The particle size appeared much larger than the measured particle size, possibly attributable to the agglomeration and coalescence of the particles when water was removed through either air drying or the vacuum system of the SEM before imaging.

MC-FD showed a clustered and continuous network with many heterogeneous voids, and the particles come in a range of different sizes. In addition, the particles were agglomerated and adhered to each other. It possesses an irregular shape, flake-like and with a highly porous structure [42,43]. The formation of the porous structure in the MC-FD was caused by removing water and sublimating the ice crystals during the primary drying cycle in freeze drying [44,45]. In addition, the samples collapsed because of structural transformations caused by temperature and water content changes that occurred during the lyophilisation process [46]. During freezing, the emulsion was susceptible to instability induced by ice formation, fat crystallisation, interfacial transitions, freeze-concentration, and biopolymer conformational changes [47]. Ice crystal development promotes particle aggregation that induces disruption of the interfacial layer and inter-droplet emulsifier interactions induced by insufficient unfrozen water being present to fully hydrate the emulsifier molecules adsorbed to the droplet surfaces [47]. Eratte et al. [42] and Yazicioglu et al. [33] reported similar results where the MC-FD was more irregularly shaped and highly porous. The authors also mentioned that the porous structure might lead to further oil leakage, resulting in a higher surface oil content.

### 2.3. Thermal Behaviour of CV-E and MC-FD

**Figure 2:**
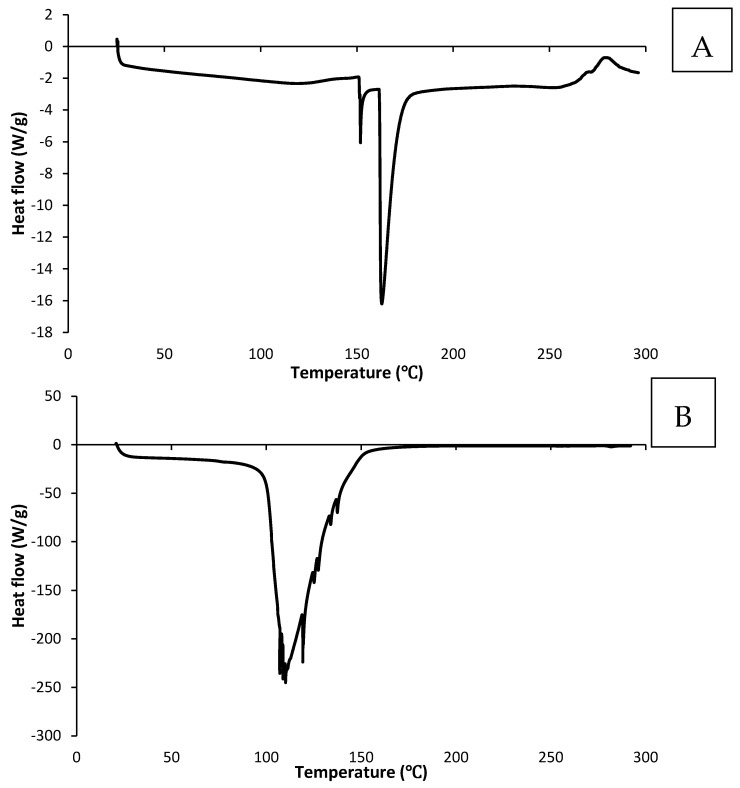
DSC melting curves of MC-FD (**A**) and CV-E (**B**).

Three thermal events were observed in MC-FD (Table 3). The first tiny peak had a melting point of 151.63 °C followed by a large and sharp second peak at 161.77 °C, related to the thermal degradation and polymer decomposition of the combination of wall materials and core materials [48]. The endothermic peak for the first and second peaks could also be associated with the cleavage of the electrostatic interactions between the two oppositely charged wall materials [49]. The third peak represents a small exothermic peak at 268.42 °C, which could be associated with crystallisation, oxidisation, or decomposition reactions [50]. The material decomposition was caused by the pyrolysis of polysaccharides, starting with a random split of the glycosidic bonds and proteins [51]. The characteristic curve of a physical mixture is generally comprised of peaks related to the polymer utilised as encapsulating wall material and to the encapsulated compound, as seen in Figure 2A. The DSC curve of MC-FD only displayed a large and sharp endothermic peak of the wall materials without the core material’s melting peak. Similar results were reported by Rutz et al. [50] and Sansone et al. [52], in whose work the melting curve of the core material was absent due to the successful entrapment of the core materials.

The DSC of the CV-E (Figure 2B) exhibited a large endothermic peak with a melting point of 105.99 °C. The peak corresponds to the loss of water (both free and bound molecules) mainly associated with the surrounding water system and some from the hydrophilic groups of the polymer and the dehydration of the polymeric surface of the emulsion [53]. No other peaks were observed, indicating that the emulsion peak is likely covered up by the large peak caused by the evaporation of water. This also shows that the CV-E has a much lower thermal stability than MC-FD, as MC-FD has a much higher endothermic peak, and the wall material and core material could withstand the heating process of up to 160 °C.

### 2.4. FTIR of Lipid Particles

**Figure 3:**
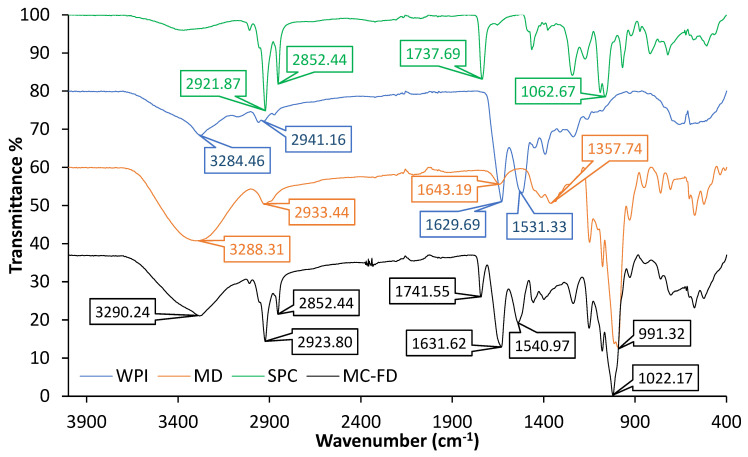
FTIR spectra of WPI, MD, SPC, and (MC-FD).

The spectrum of SPC displayed characteristic peaks at 2921.87 cm^−1^ and 2852.44 cm^−1^ associated with –CH stretching, as shown in Figure 3. The strong transmittance band at 1737.69 cm^−1^ corresponded to the characteristic bond of the ester carbonyl functional group of the phosphatidylcholine [54]. A broad transmittance band was observed around 3288.31 cm^−1^ for MD, which is related to –OH stretching groups, and the absorbance at 1643.19 cm^−1^ is the asymmetrical and symmetrical stretches of –C=O and O–H bending at 1357.74 cm^−1^. The most distinctive spectral features of WPI are the amide I and II bands [55]. WPI presented strong amide-type I at 1629.69 cm^−1^, amide II C-N stretching at 1531.33 cm^−1^, and results are in agreement with Hosseinnia et al. [56]. Shifts of the amide I and amide II bands indicate changes in protein secondary structures such as hydrogen bonds, disulphide bonds, etc. [57]. The results showed strong inter- and intra-molecular hydrogen bonds (O–H stretching) at 3284.46 cm^−1^ and high hydrophobicity (C–H stretching) at 2941.16 cm^−1^. These spectrum results were similarly reported by Chew et al. [58] and Karrar et al. [25], who also utilised the same wall materials for encapsulation.

The characteristic peaks of the wall materials and core materials were all observed in the spectra of MC-FD. This indicates no significant interactions or modifications between the core and wall materials. The results demonstrated that SPC was successfully encapsulated in the microcapsules, and its structural integrity was retained with efficient chemical stability.

### 2.5. In Vitro Digestion of Lipids

**Figure 4:**
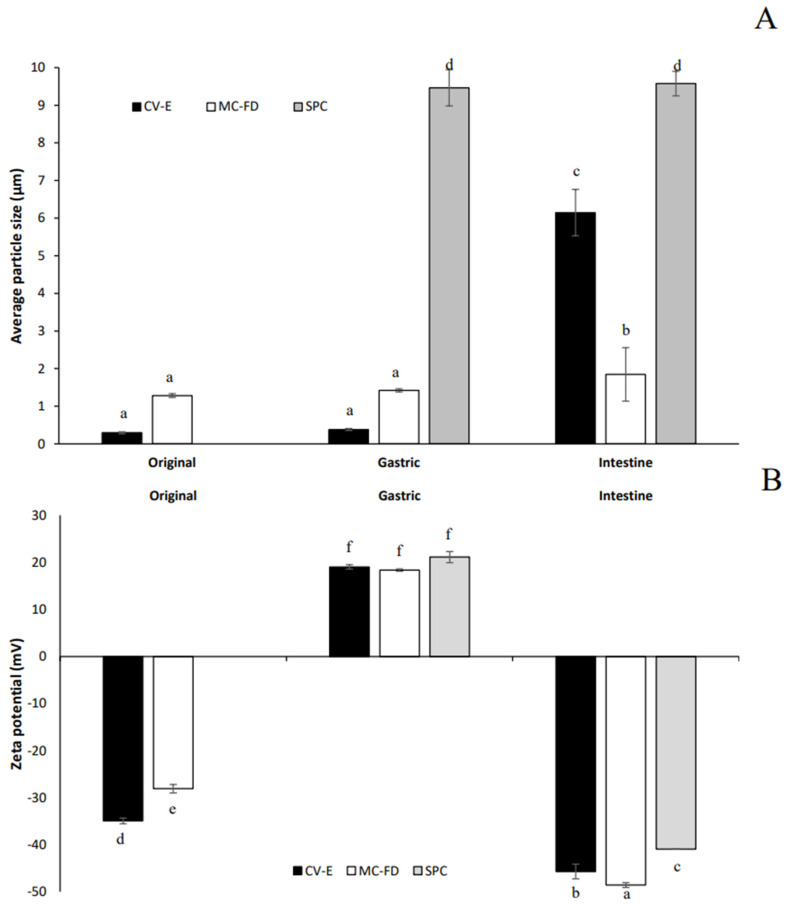
Average particle size D_3,2_ (**A**) and zeta potential (**B**) of CV-E, MC-FD, and SPC. Error bars represent means (n = 3) ± standard deviations. Different letters indicate whether they are significantly different in the original form, gastric, and intestinal phases (Duncan, *p* < 0.05).

A simulated in vitro digestion model consisting of the gastric and intestinal phases was utilised to investigate the mean particle size, particle size distribution, zeta potential, and microstructure of the SPC, CV-E, and MC-FD. The original average surface weighted mean particle size of the CV-E was particularly small at 0.295 µm, but the MC-FD was significantly (*p* < 0.05) larger at 1.28 µm, as observed in Figure 4. After passing through the gastric phase, both CV-E and MC-FD displayed no significant increase (*p* > 0.05) in particle size. However, other studies by Zhang et al. [59] and Zhang et al. [15] reported contrasting results in which a significant increase in average particle size for the gastric phase was observed. This shows that CV-E and MC-FD are incredibly stable and resistant to droplet aggregation under gastric conditions, even though other studies have reported that protein-stabilised emulsions are susceptible to aggregation under gastric conditions. The type of emulsion instability that transpires could be due to the hydrolysis of adsorbed proteins by pepsin, which reduces droplet stability to coalescence, and changes in pH and ionic strength that may promote aggregation caused by the weakening of the electrostatic repulsion between lipid particles [59,60]. An explanation could be SPC’s amphiphilic properties, which could act as a surfactant or emulsifier to inhibit particle aggregation.

After they had passed through the small intestinal phase, a significant increase (*p* < 0.05) in average particle size was observed for both CV-E and MC-FD. The increase in size was drastically more pronounced compared to samples previously digested in the gastric phase. The increase in particle size is possibly due to particle aggregation that may have induced several physiochemical mechanisms. The transportation of the emulsions from the stomach to the small intestine would involve an increase in pH, which could have promoted aggregation in the WPI-MD-coated particles as they moved through their isoelectric point [61]. The addition of inorganic salts could also have promoted particle aggregation by screening the electrostatic repulsion between the particles [62]. In addition, the bile salts could have been adsorbed to the particle surfaces and altered their interfacial compositions, thereby altering particle–particle interactions [63]. After intestinal digestion, a significant increase (*p* < 0.05) in particle size from 0.381 µm to 6.15 µm was observed in CV-E. These destabilisation mechanisms could be promoted due to the changes in the composition and properties of the lipid particle surfaces within the GIT due to displacement or digestion of the original emulsifiers [60]. In addition, the lipase activity at the oil droplet surface can also alter interfacial properties, leading to a decrease in coalescence stability [64]. Other studies have reported similar results, with a significant increase in particle sizes after intestinal digestion. MC-FD has no significant increase (*p* > 0.05) in particle size from gastric digestion, with only a slight increase from 1.42 µm to 1.85 µm. This indicates that the MC-FD possesses superb stability and provides protection for the core material even under the harsh conditions of the digestion process.

The original CV-E and MC-FD demonstrated relatively good stability with negative charges of −34.9 mV and −28.1 mV, respectively. The high negative charge is primarily attributed to the whey protein molecules at the particle surfaces. The magnitude of the electric charges may also be caused by anionic or cationic impurities in the oil phase that could adsorb to the oil–water interface and contribute to the overall interfacial charge, including FFAs, PLs, or mineral ions [15]. Moreover, WPI is above its isoelectric point in the original solution conditions (pH 7), giving a negative charge [15].

A drastic change in zeta potential to +19.07 mV and +18.38 mV was achieved when the CV-E and MC-FD were subjected to simulated gastric conditions, respectively. This is because the simulated gastric fluids have a low pH and high ionic strength, resulting in a significant change in the electrical properties of the particles due to electrostatic screening effects [62]. The low pH during gastric digestion indicates that the whey proteins in the samples would become negatively charged as they are below their isoelectric point. However, the results show that the zeta potential obtained is positive, implying that the whey protein could have been digested and displaced [15]. The surface layers of these particles were affected by digestive enzymes, and some negative charge from the residues in the protein interface was lost [65]. As a result, the electrostatic repulsion between the particles decreased, leading to their aggregation.

After the intestinal digestion, the CV-E and MC-FD have high negative charges of −45.7 mV and −48.58 mV, respectively. MC-FD showed a slightly significant difference in zeta potential which was higher than that of CV-E, indicating better stability due to higher electrostatic repulsion. However, the high negative charge obtained could be attributed to the presence of anionic species such as undigested lipids, undigested proteins, vesicles, micelles, and calcium salts. These could originate from the CV-E and MC-FD (peptides, FFAs, or PLs) or the simulated gastrointestinal fluids (bile salts and PLs). The anionic bile salts could displace the original emulsifier molecules from the surfaces, thereby modifying their surface charge. Moreover, the hydrolysis of lipids by pancreatic lipase at the surface of the lipid articles can cause the release of anionic FFAs, which also alters the surface charge. As these particles could contribute to the overall electrical signal utilised to calculate the electrophoretic mobility, the results should be treated with some caution [15]. Most studies report similarly high negative charges after the intestinal phase [15,61].

**Figure 5:**
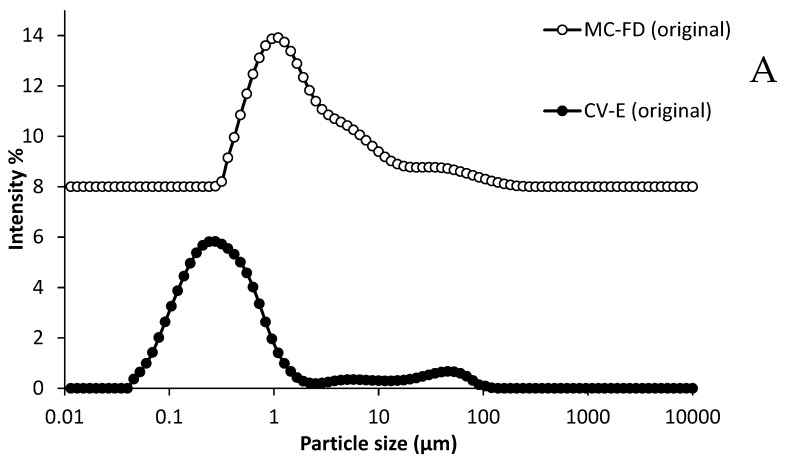

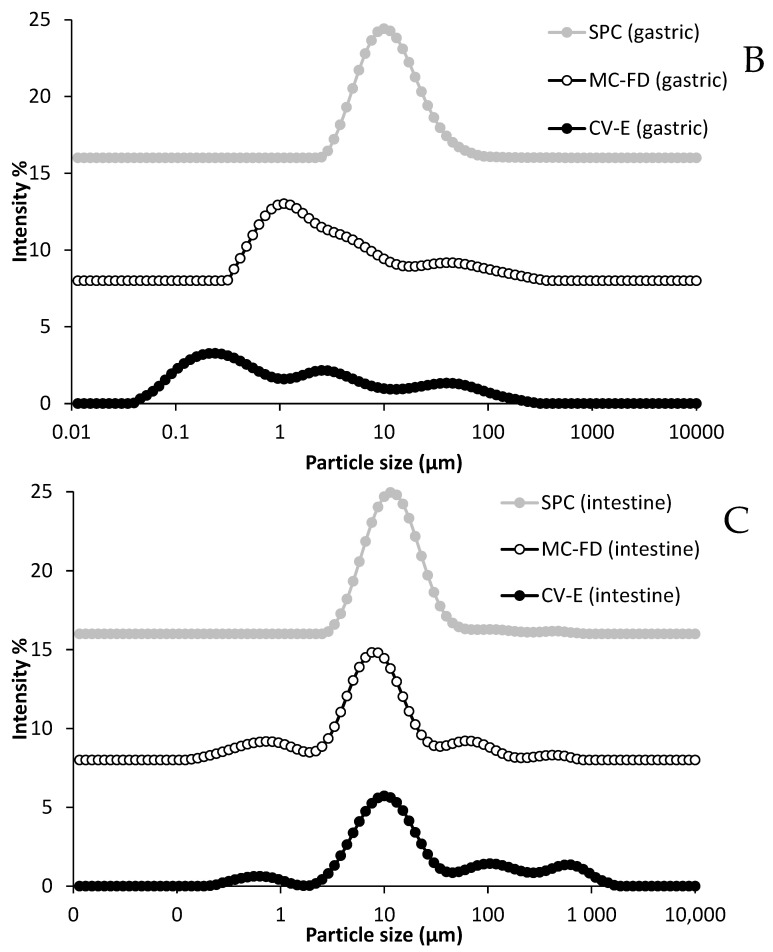
The particle size distribution of CV-E, MC-FD, and SPC after in vitro digestion in the original (**A**), gastric (**B**), and intestinal phases (**C**).

The original phase shows that both CV-E and MC-FD have a monomodal size distribution (Figure 5A). After gastric digestion, the size distribution shifted toward larger sizes and the peak became broader, possibly due to the reduced droplet stability caused by the coalescence and aggregation of particles initiated by the hydrolysis of adsorbed proteins and changes in pH and ionic strength [60]. The gastric digestion of CV-E presented a polydisperse distribution with three broad conjoint peaks ranging from 0.04 to 275 µm. The MC-FD displayed a slightly sharper peak ranging from 0.3 to 363 µm with a bimodal size distribution. There was also no significant shift in the peak except for a slight decrease in the intensity of the first peak. This shows that MC-FD provided better protection than the CV-E as there was a larger shift in the CV-E peak compared to MC-FD.

For intestinal digestion, the particle size distributions for both CV-E and MC-FD are unimodal and similar, with increasing detection of particles in the size ranges of 0.15 to 724 µm (MC-FD) and 0.24 to 1659 µm (CV-E). This is possibly caused by the formation of micelles and vesicles containing lipolytic products [66]. A further shift in the size distribution towards larger sizes was in agreement with the average particle size results. This is because the surface-active species, including enzymes and bile salts, and the changes in pH and ionic strength in the simulated intestinal fluid led to aggregation and coalescence [67].

After gastric and intestinal digestion, it was observed that SPC was largely unaffected by the digestion process. The digestion of SPC displayed no significant difference (*p* > 0.05) in particle size, and it demonstrated a monomodal size distribution in the gastric and intestinal phases from approximately 2 to 91 µm. There was also no shift in the particle size distribution after the gastric and intestinal phases. This is due to the ability of PLs to form micelles in a hydrophilic system that protects it from the harsh conditions of digestion. However, there was a significant change (*p* < 0.05) in the zeta potential of SPC, largely because the electric properties are influenced by the simulated gastric and intestinal fluid conditions (pH and ionic strength). As SPC does not dissolve in water solution, the original SPC particle size and zeta potential analysis were omitted.

**Figure 6:**
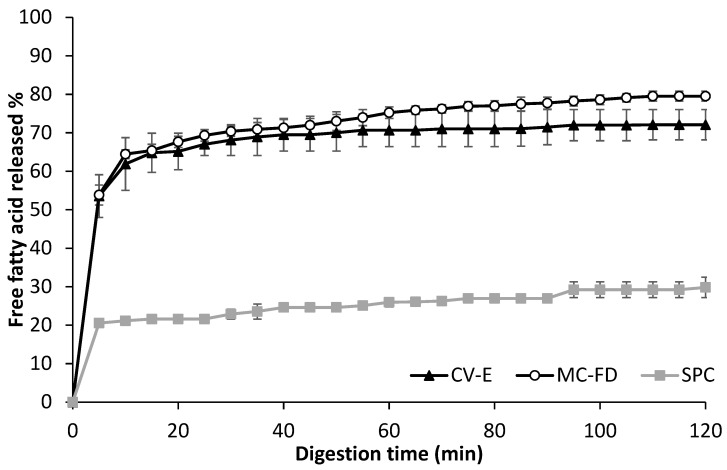
% of FFAs released in CV-E and MC-FD measured with a pH-stat in an in vitro digestion model. Data points and error bars represent means (n = 2).

Measurement of lipid digestibility is carried out through the release of FFA as it allows us to determine the capability of these systems to protect the emulsified lipid against lipolysis. All samples had an initial increase in digestibility, possibly caused by the digestion of proteins, PLs, or other substances in the emulsions [15]. Similar trends were observed for the digested samples, in which an initial rapid increase in FFAs was released, followed by a gradual increase as digestion time prolonged until a constant final value was obtained. This is due to the inhibition of lipase activity from pancreatic lipase that prevents access to the lipid core because of the accumulation of lipolytic products at the interface, such as fatty acids [66,68]. However, the FFA released could be accelerated in the presence of higher calcium concentrations [69]. This is because calcium combines with long-chain fatty acids to form insoluble soaps, which precipitate from the oil–water interface that enhances the access of pancreatic lipase to the lipid core [70]. A slight difference in the digestibility of the lipids in CV-E and MC-FD can be seen in Figure 6. No significant differences in the FFA released were observed initially. However, as it reached 45 min of digestion time, MC-FD exhibited a slightly higher increase in FFA released, reaching 79.54% at 120 min, while CV-E had 72.1% FFA released. A possible explanation could be the aggregation and coalescence of the CV-E shown in Figure 5A, and even the colloidal-forming properties of SPC that prevented the lipase and bile salts from accessing the encapsulated lipid due to the decrease in the surface area of the lipid droplets [61]. The fact that there was no significant difference in the digestibility between the CV-E and MC-FD suggests that the lipase could readily access both encapsulated lipid samples. Previous studies have suggested that adding bile salts to PC-stabilised emulsion disrupted the PL’s packing, forming a mixed PL/bile interface. This promoted the pancreatic lipase activity, as lipase further adsorbed to the surfaces of droplets coated with either PL or bile extract [71]. The results show that the digestion of two different forms of encapsulation, CV-E and MC-FD, may have slightly affected the lipids’ digestibility.

On the contrary, the lipid digestibility for SPC was considerably lower than CV-E and MC-FD, with a sharp increase in the first 5 min and a gradual increase until it reached 29.82% FFA released at 120 min of digestion time. Zhang et al. [15] explained that the SPC could not have been well digested by the porcine lipase utilised in the simulated in vitro digestion model in this study. It could also be the unique properties of SPC as a PL to form colloidal structures that may have been resistant to intestinal digestion by inhibiting the lipase from accessing the encapsulated lipid. Furthermore, digestion products accumulated in the reaction mixture while the reactants consumed were not replenished. This resulted in bile salt micelles becoming saturated with fatty acids and monoglycerols, minimising their ability to clear fatty acids off the interface.

### 2.6. Microstructure of CV-E and MC-FD During Digestion

**Figure 7:**
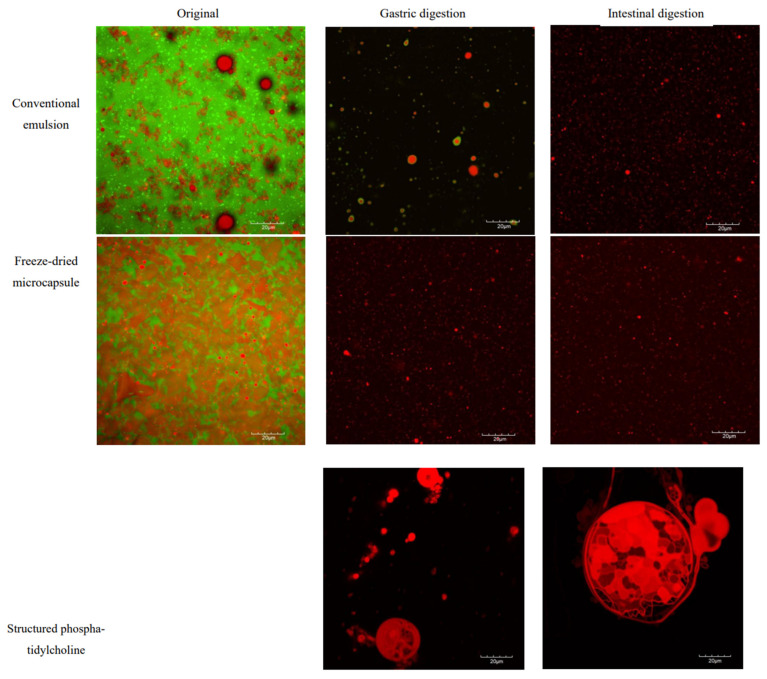
Microstructure of CV-E, MC-FD, and SPC after exposure to simulated gastric and intestinal digestion.

The original CV-E exhibited nano-sized emulsion with flocculation of the particles (Figure 7). This phenomenon probably occurred as droplet aggregation on heating causes non-covalent interactions between unfolded protein molecules adsorbed on different particles or depletion flocculation caused by non-adsorbed protein aggregates [72]. A study by Ye et al. [73] produced an emulsion using WPI as an emulsifier and reported similar emulsion particle flocculation before digestion. Black spots (MD) overlap the red emulsion (lipid) of the CV-E, indicating the lipids were encapsulated within the MD and WPI wall matrix. For the original MC-FD, the images show that the microcapsule appears flat and irregularly shaped with some droplets of lipid emulsion.

After gastric digestion, there was a shift in CV-E particle size distribution to the higher particle size range, resulting in three broad peaks. This indicates that slight flocculation and coalescence of the emulsion droplets occurred due to the action of pepsin. The CLSM image of CV-E after gastric digestion illustrates a stable emulsion with a green layer of WPI encapsulating the lipid. This shows that the lipid core is still well protected within the wall materials, and the results are in agreement with the obtained particle size, showing no significant change in the average particle size. In addition, the zeta potential of the samples is relatively high at approximately +20 mV, which may be why no extensive aggregation and coalescence occurred in the CLSM images. Another feasible explanation could be the smaller particle sizes of the original CV-E shown in Figure 4, which leads to higher stability against gravity due to Brownian motion, as the particles were moving around rapidly during the capture of the CLSM images [74]. However, there was no WPI in the gastric digestion of MC-FD, and only lipid droplets were present after gastric digestion. This indicates that the protein was completely digested after gastric digestion as it has poorer stability, possibly due to its irregular shape, and a highly porous structure that may lead to further leakage of the core material [42]. This could be part of the reason why MC-FD has a higher FFA release than CV-E. The results obtained in this experiment are in contrast to other studies that displayed extensive droplet aggregation and coalescence after gastric digestion [15,61,73,75].

After the intestinal digestion of the samples, a wide range of particle sizes, including relatively large particles, was detected with CLSM. The slight increase in larger particle sizes suggests that the oil phase was not being digested, but rather that certain droplet coalescence may have occurred in these emulsions once the initial protein emulsifier layer was digested and displaced in the small intestinal phase [76]. However, there was also the breakdown of the lipid droplet aggregates and stabilisation of the lipid droplet occurring, caused by the adsorption of negatively charged bile salts at their surface, providing electrostatic repulsions between particles [66]. This was supported by the high zeta potential of the samples at approximately −40 mV to −48 mV, and the images seen in both samples show that the particles were not aggregating. The CLSM images of CV-E and MC-FD after intestinal digestion were relatively the same, except that there were additional large lipid droplets, as shown in the average particle size and the particle size distribution.

After gastric and lipid digestion, the SPC images show that the lipid particles were considerably aggregating and coalescing. The particles observed were coalescing into a large emulsion droplet with small emulsion droplets inside after gastric and intestinal digestion. Based on previous analysis, no significant differences were observed in the particle size, size distribution, and FFA released. Therefore, the coalescence likely occurred before gastric digestion, resulting in no significant change in particle size and size distribution between gastric and intestinal digestion. As previously mentioned, the reason was the ability of PLs to form micelles, as seen in the images. This prevents the lipase from accessing the lipid particles; hence, the FFA released was significantly lower compared to the lipid samples.

### 2.7. Fatty Acid Composition After Digestion

Table 4 presents the fatty acid compositions of CV-E, MC-FD, and SPC before and after digestion. Linoleic acid (C18:2), primarily contributed by soybean oil, was the predominant fatty acid in both CV-E and MC-FD formulations, followed by NA (C24:1) from SPC. In contrast, SPC exhibited a higher proportion of NA, with linoleic acid being the second most abundant fatty acid. Following digestion, there was a noticeable reduction in total saturated (SFA) and polyunsaturated fatty acids (PUFA), particularly palmitic acid (C16:0), stearic acid (C18:0), oleic acid (C18:1), linoleic acid (C18:2), and linolenic acid (C18:3). This is likely due to the preferential binding of lipase to shorter-chain fatty acids and its affinity for the oil–water interface, which promotes faster hydrolysis of more accessible fatty acids [60]. Similar reductions in SFA and PUFA after digestion have been reported in previous studies [77,78,79]. Additionally, oxidative degradation may have contributed to the significant loss of PUFAs in both CV-E and MC-FD, particularly after gastric digestion.

A significant increase in MUFA was observed in CV-E after digestion, primarily attributed to the relative enrichment of nervonic acid (NA). However, this increase does not reflect enhanced release of NA; rather, it results from the preferential hydrolysis of shorter-chain fatty acids, which lowers their relative proportion. Due to NA’s long-chain structure and high hydrophobicity, it is less accessible to digestive enzymes, hindering its hydrolysis and subsequent absorption in the small intestine [80]. Several mechanisms may explain this limited digestibility. First, the amphiphilic nature of structured phosphatidylcholine (SPC) may facilitate micelle formation, sequestering NA within the matrix and reducing lipase accessibility. Second, calcium ions present in the simulated digestive fluids may interact with NA released from SPC lipolysis to form insoluble calcium soaps, which can precipitate onto emulsion droplets and further reduce NA bioaccessibility [81]. Third, surface-active emulsifiers—such as small-molecule surfactants, phospholipids, and proteins—can interfere with lipase binding at the oil–water interface by altering interfacial properties and thereby decreasing hydrolytic efficiency [60]. The primary fatty acids digested in both formulations were oleic acid and linoleic acid, which are shorter in chain length and present in higher concentrations. Their physicochemical properties make them more prone to lipase-mediated digestion, in contrast to NA which remains largely undigested.

Interestingly, MC-FD showed a greater proportional reduction in NA (42.39%) compared to CV-E (45.39%). This suggests that the freeze-drying process may have altered the structural integrity of the encapsulation matrix—as supported by the SEM imaging in Figure 1—leading to changes in the release dynamics and overall digestibility of NA [82]. Such structural modifications may enhance the exposure of NA at the oil–water interface or within the matrix, facilitating slightly improved bioaccessibility. Additionally, a significant reduction in SFA was observed in CV-E, while MC-FD showed a more pronounced decrease in MUFA, particularly NA. This pattern implies that specific fatty acids may migrate to the oil–water interface during digestion, increasing their susceptibility to enzymatic hydrolysis. This migration could provide insight into which fatty acids are preferentially released depending on the structure and composition of the delivery system. These observations highlight the importance of matrix structure, enzyme accessibility, and interfacial characteristics in modulating NA release. They underscore the need for tailored delivery strategies to improve the release and bioavailability of hydrophobic, long-chain fatty acids.

### 2.8. Primary and Secondary Oxidative Stability of Lipid Particles

PV is utilised as a standard index to monitor the quality of lipids by measuring the content of hydroperoxides in lipids [48]. Hydroperoxides have no colour and odour but are highly toxic and can hinder the bioavailability of fatty acids [83]. Therefore, the oxidative stability of CV-E and MC-FD was studied with the setup of a storage trial (4 °C, 25 °C, and 45 °C at 75% relative humidity) for up to 90 days to mimic real-world applications of sample storage. A storage temperature of 45 °C was used as an accelerated storage stability test.

**Figure 8:**
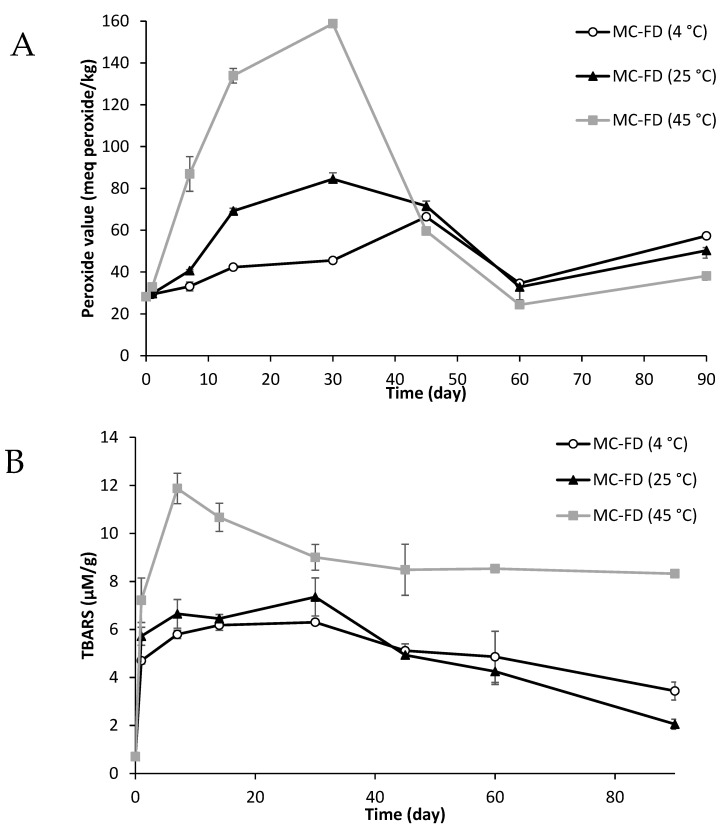

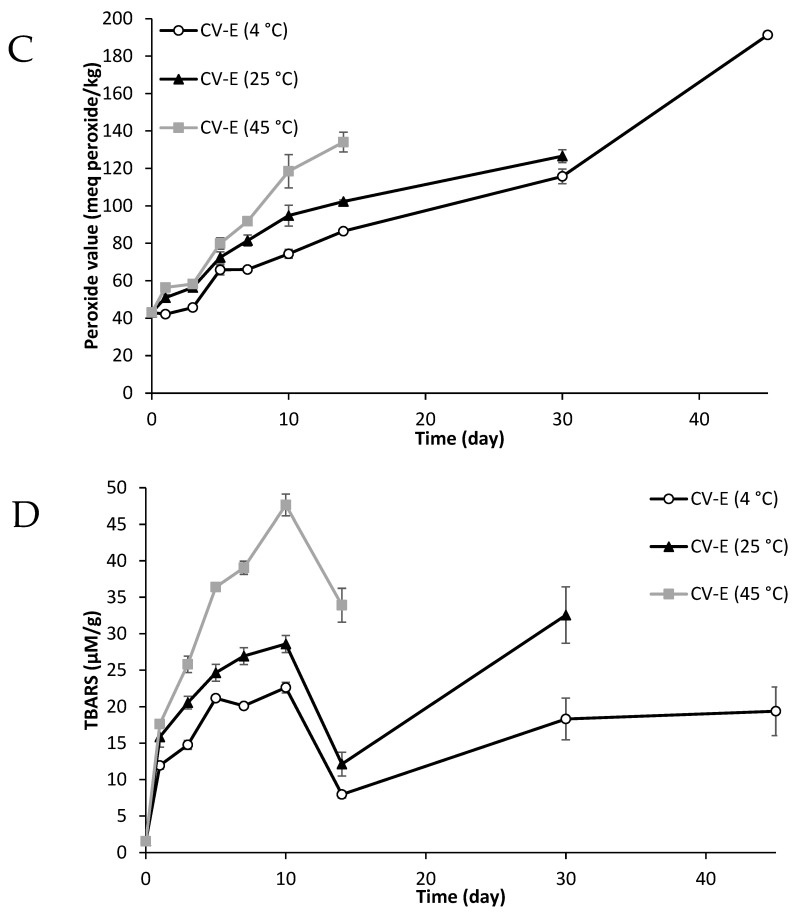
Formation of lipid hydroperoxides and thiobarbituric acid reactive substances (TBARS) in MC-FD (**A**,**B**) and CV-E (**C**,**D**) during storage up to 90 days at 4 °C, 25 °C, and 45 °C. Data points and error bars represent means (n = 2) ± standard deviations.

As shown in Figure 8A, the PV of MC-FD drastically increases as temperature rises. MC-FD (45 °C) has a sharp increase in PV, reaching a maximum of 158.78 ± 0.8 meq peroxide/kg on day 30 before decreasing to 24.34 ± 1.76 meq peroxide/kg on day 60. Similar trends were observed in MC-FD (4 °C and 25 °C), except that the increase in PV for MC-FD (4 °C) was significantly lower than MC-FD (25 °C). At low temperatures (4 °C), the reaction rate and thus the oxygen consumption rate is slow, and oxygen availability is not rate limiting. However, the reason for the decrease in oxidative stability of MC-FD at higher temperatures could be the increase in permeability, porosity, and the surface fat of wall material [84,85]. This is because the increase in temperature led to an increase in permeability caused by the alteration in glass transition temperature that decreased the stability of the structure [86]. The oil was released to the surface of the powder during storage under high temperatures due to physical and chemical changes in the wall materials and the molecular diffusion of the oil through them [87]. Therefore, proteins denature at high temperatures during storage, which could lead to lower protection properties of protein wall materials due to the changes in their structure. The PV of MC-FD (4 °C) remained relatively stable, with only a slight increase in PV until it reached a maximum of 66.42 ± 0.29 meq peroxide/kg on day 45. PV subsequently decreased on day 60 for MC-FD (4 °C), while the PV of MC-FD (25 °C and 45 °C) decreased on day 45 due to decomposition of peroxide as previously mentioned. The rapid increase followed by a fast drop in PV for MC-FD (45 °C) has been previously reported in other oil-in-water emulsion systems [88,89].

Figure 8C shows that CV-E had an initial slow increase in PV from day 0, followed by a sharp increase on day 5 and another large increase on day 10. CV-E (4 °C) remained stable until day 3, before a continued increase up to a maximum of 191.26 ± 1.95 meq peroxide/kg. CV-E (25 °C and 45 °C) had a much higher increase in PV and became a solid that could not be extracted for PV measurement on day 45 and day 30, 45, respectively. This shows that CV-E has poor stability that results in a sharp increase in PV in a relatively short time. This is because, in a liquid matrix, reactants of lipid oxidation (such as oxygen) have higher molecular mobility and thus can interact to a greater extent [90]. As water plasticises hydrophilic macromolecules, the increase in matrix molecular mobility is likely to increase diffusion within the matrix [91]. Transition metals may be attracted to the droplet surface if it is negatively charged. This can contact lipid hydroperoxides in the oil, as these are often surface-active and thus tend to accumulate at the phase boundary in emulsions [92]. Transition metals such as copper and iron catalyse the decomposition of lipid hydroperoxides into very reactive alkoxyl and peroxyl radicals, which may then react with surrounding unsaturated lipids [93]. The lipid phase in CV-E has a high content of unsaturated fatty acids, as soybean oil was used as a dispersion for SPC, which may be why there is a high initial rate of oxidation.

The PV for the first 5 days of CV-E increased sharply on day 3. However, on day 0, CV-E had a significantly higher PV of 43.1 meq peroxide/kg compared to MC-FD, which had a PV of 28.25 meq peroxide/kg, even though MC-FD was obtained by freeze-drying of the CV-E sample. Kumar and Kalonia [94] reported that freeze-drying decreased the level of peroxides present in polyethene glycols. This could be why MC-FD had a lower amount of PV than CV-E.

The results showed that the freeze-drying process provided more effective protection against oxidation during storage than CV-E. The samples, especially MC-FD, exhibited lower PV during storage. The results agree with Partanen et al. [91], who reported that flaxseed oil encapsulated with WPI as a wall material effectively delayed the oxidation process. Charoen et al. [95] reported that the higher oxidative stability of encapsulated peony seed oil was contributed by the antioxidative properties of WPI, which contains acidic amino acids and shows metal binding mechanisms through the involvement of carboxyl groups of aspartic and glutamic acids. Wang et al. [96] also mentioned that this might be due to the presence of SPC, which effectively delayed the oxidation process in encapsulated lipids during the storage period. This is because the high antioxidant effect of SPC is attributed to PLs, which can offer an oxygen barrier effect at the oil/water interface and provide a protective barrier for lipids against atmospheric oxygen [97]. The decrease in all samples may be attributed to the encapsulated SPC. PLs may exert an antioxidative effect through proposed mechanisms, including the decomposition of hydroperoxides, chelation of metals, formation of Maillard reaction products, and formation of oxygen barriers [97,98].

Secondary products of oxidation are formed from the breakdown of hydroperoxides. TBARS measures saturated aldehydes, 2-enals, and 2-dienals produced in the termination phase of lipid peroxidation [99]. TBARS was selected for the secondary oxidative test because it is one of the most frequently used tests for assessing lipid oxidation and it can be easily compared with other studies that have been carried out on lipid encapsulation.

MC-FD on day 0 had a very low TBARS value of 0.7 µM/g of lipid and started increasing as temperature and storage increased (Figure 8B). The TBARS values for MC-FD (45 °C) sharply increased, reaching a maximum of 11.87 µM/g at day 7. The TBARS values for MC-FD (45 °C) then subsequently decreased to approximately 8.5 µM/g at day 45 and remained the same from day 45 to 90. The MC-FD (4 °C and 25 °C) samples had a similar trend, with a sharp increase on day 1 followed by a gradual increase, and started to decrease from day 30. As previously mentioned, studies have reported that WPI has antioxidant activity that may have contributed to the decrease in TBARS value [100,101]. A study by Charles et al. [102] reported similar TBARS values on day 0 at 25 °C using the same wall materials for microcapsules but failed to inhibit oxidation after 15 days, which steadily rose to day 90 of the storage stability test. However, in this study, MC-FD displayed superior stability against secondary oxidation, in which the TBARS value remained low throughout storage at 4 °C, 25 °C, and 45 °C.

In this study, TBARS values of MC-FD were mostly below 10 µM/g throughout storage. Song and Shurson [103] mention that a low TBARS value could result from aldehydes that have not yet been produced or volatile aldehydes that have already been lost during the processing and storage of the lipid. In addition, several non-peroxidation substances, such as soluble proteins, peptides, and amino acids, can interfere with the TBA reagent and give false readings [104]. Montero et al. [105] have also reported an increase in TBARS value and subsequent decrease during storage. MC-FD (45 °C) had a significantly higher TBARS value compared to the storage temperatures at 4 °C and 25 °C due to the rupture of the wall materials at high temperatures. This was supported by a study by Jia et al. [106], who reported that there were different degrees of rupture for the microcapsule wall, and higher temperatures led to more severe ruptures. This resulted in more lipid migration to the solid–air interface, which reduced the stability and contributed to the increment of lipid oxidation.

For all samples under storage, the TBARS value rose according to temperature and storage time. The TBARS value in CV-E rapidly rose to day 10 before quickly decreasing on day 14. CV-E (4 °C and 25 °C) subsequently increased up to day 30, and CV-E (4 °C) remained the same till day 45. By comparing CV-E and MC-FD, it was observed that TBARS values were higher in CV-E. This was expected as previously mentioned, CV-E has higher molecular mobility for reactants of lipid oxidation. The increase in matrix molecular mobility is likely to increase diffusion within the matrix, and the solubility of oxygen may be influenced by an increase in water content [90]. Another reason MC-FD tends to have lower oxidation than CV-E is because of the wall materials that protect the lipids from exposure to oxygen. Some polysaccharides, such as the MD used in MC-FD and CV-E, have been claimed to have antioxidant activity due to their ability to donate hydrogen and act as radical chain breakers [92]. In addition, globular proteins such as WPI have also been reported to inhibit lipid oxidation due to free radical scavenging by sulfhydryl and non-sulfhydryl amino acids, plus some limited transition metal chelation [107]. The antioxidant effects may have caused a reduction in the TBARS value of these samples, and it is possible that when their effects ran out, the TBARS value started increasing again.

EE plays a crucial role against lipid oxidation, as the increased EE decreases lipid oxidation by reducing free oil content [108]. Other physicochemical factors may also affect the oxidation rate, depending on the type of interfacial coatings formed by different wall materials. This is because they have different abilities to prevent transition metals from reacting with the emulsified lipids due to factors such as thickness, packing, or chemical composition. Carneiro et al. [109] and Ramakrishnan et al. [110] investigated the performance of selected wall materials against lipid oxidation of encapsulated flaxseed oil and fish oil. The authors reported that the combination of WPI and MD had the highest protection against lipid oxidation. This was attributed to the combination of the antioxidant activity of WPI and the molecular weight of the materials conforming to the microcapsule wall.

### 2.9. Particle Size and PDI of Lipid Particles During Storage

A narrow particle size distribution minimizes the concentration gradient to the environment and inhibits the Ostwald ripening process (mass transfer from small particles to larger particles) [111]. Hence, monitoring the changes in particle size during a storage trial is the best method to determine the physical stability of the system [112].

**Figure 9:**
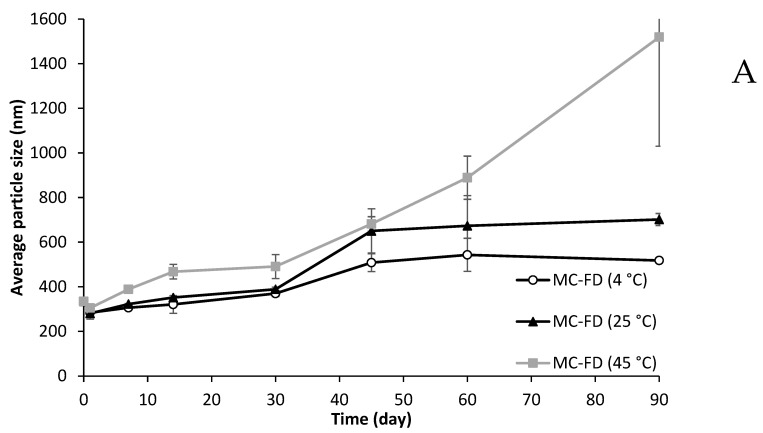

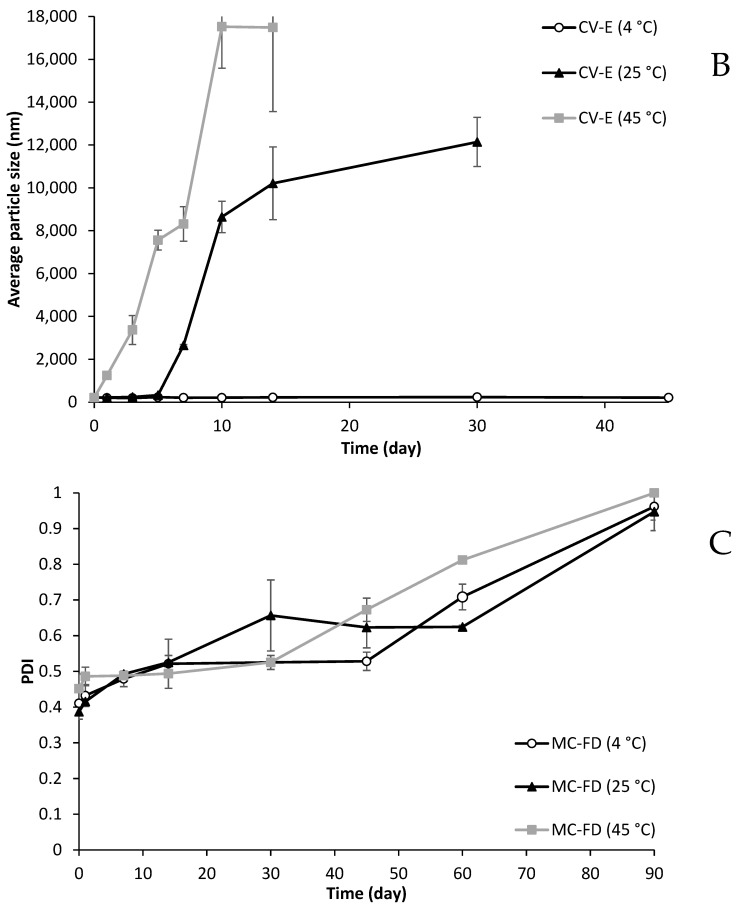

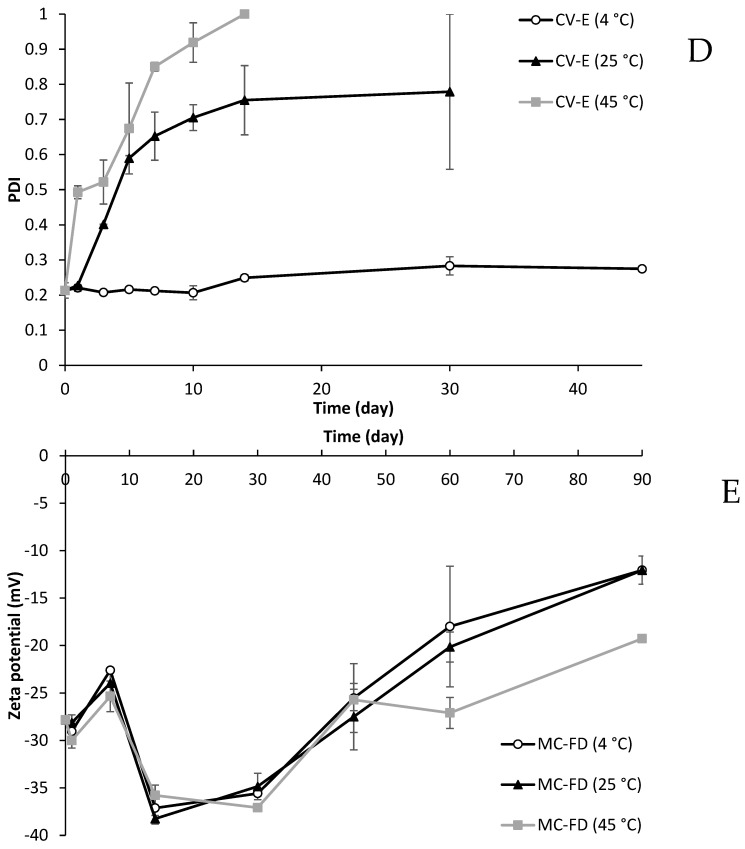

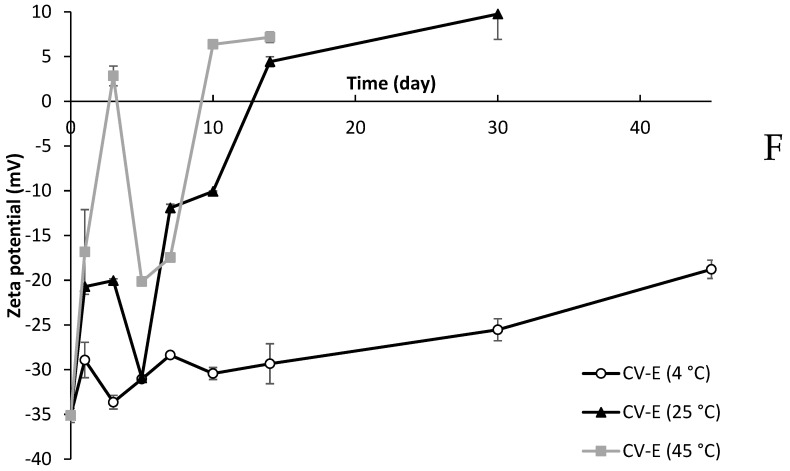
MC-FD and CV-E average particle size (**A**,**B**), PDI (**C**,**D**), and zeta potential (**E**,**F**) during storage at 4 °C, 25 °C, and 45 °C for up to 90 days, respectively. Data points and error bars represent means (n = 2) ± standard deviations.

The average particle size of MC-FD showed an upward trend during a storage trial of up to 90 days (Figure 9). The temperature at 4 °C and 25 °C displayed a relatively stable particle size of 280 nm from day 1 to day 14 and gradually increased to 388 nm on day 30. On day 45, the average particle size started to elevate slightly for 4 °C and 25 °C, and remained stable until day 90. However, the temperature of 45 °C demonstrated a constant increase in particle size, reaching over 1000 nm. This is because of the high kinetic energy at high temperatures that accelerated the collision of the nanoparticles, thereby increasing the probability of agglomeration between nanoparticles [113]. The standard deviation of MC-FD at 45 °C also increased drastically towards days 60 and 90 due to the destabilisation mechanism mentioned earlier that led to the severe aggregation of particles. The aggregation of particles can negatively affect the PDI as the particle size distribution becomes non-uniform, which was observed in MC-FD at all temperatures. The PDI of MC-FD increased appreciably to 1 or close to 1 at day 90 for all temperatures, meaning that the particle size distribution is non-homogenous, likely due to caking. This shows that storage time significantly affects the average particle size and PDI of MC-FD. However, the increase in PDI was slower for lower temperatures, indicating that higher temperature affects the particle size significantly more than PDI. Furthermore, MC-FD’s particle size and PDI only remained relatively stable for up to 14 days.

CV-E at 4 °C showed incredible stability, with an average particle size of 210 nm from day 0 to day 45. PDI remained low throughout storage at around 0.2, demonstrating uniform size distribution at below 0.3 PDI. However, CV-E at 25 °C and 45 °C became unstable, with a significant increase in average particle size from 327.37 nm to 2646.5 nm (25 °C) on day 7 and 213.83 nm to 1241.67 nm (45 °C) on day 1. As expected, higher temperatures initiated an earlier onset of particle aggregation, which increased the particle sizes. As storage time increased for CV-E at 25 °C and 45 °C, particle size and PDI increased significantly, especially for CV-E at 45 °C. Emulsion and suspension are thermodynamically unstable; therefore, they are prone to breakdown over time due to various physicochemical mechanisms, including gravitational separation, flocculation, coalescence, Ostwald ripening, and phase separation [36,62]. Tse and Reineccius [114] reported that the greater instability of emulsions with increased temperature was attributable to the loss of viscosity and the increased mobility of the system. Hence, the increase in droplet size with storage time was due to the movement of the dispersed droplet through the continuous phase, hence increasing the opportunity for droplet collisions [115]. It can be concluded that the storage temperature is a crucial factor for the physical stability of emulsions and suspension.

### 2.10. Zeta Potential of Lipid Particles

The zeta potential of MC-FD at all temperatures exhibited a similar trend throughout storage, with no significant difference in zeta potential between temperatures. This indicates that temperature has no significant effect on the zeta potential. A slight decrease in zeta potential from −27.83 mV on day 0 to approximately −23 mV on day 7 was observed, before it increased again to −37 mV on day 14. Lastly, zeta potential gradually decreased to −12.07 mV (4 °C and 25 °C) and −19.27 mV (45 °C) on day 90. The gradual decrease in zeta potential throughout storage was consistent with the gradual increase in particle size of MC-FD. MC-FD had relatively good electrostatic repulsion up to day 60 before decreasing to below −20 mV. As the particles were stabilised by a combination of electrostatic (from SPC) and electrostatic (from SPC) in MC-FD, a minimum zeta potential of ± 20 mV was sufficient for particle stabilisation [112]. A similar trend was also seen in MC-FD, in which an increase in zeta potential was observed, followed by a gradual decrease over time.

Figure 9F shows that CV-E (4 °C) had the highest zeta potential, with a gradual decrease from −35.12 mV on day 0 to −18.78 mV on day 45. However, CV-E (25 °C and 45 °C) destabilised rapidly as the zeta potential dropped to less than ±10 mV by day 10. The decrease in CV-E (45 °C) was more significant, reaching a positive zeta potential of + 6.37 mV by day 10, while CV-E (25 °C) reached +4.43 mV by day 14. By day 30, the CV-E (45 °C) was immeasurable; by day 45, the CV-E (25 °C) samples became solid. The zeta potential results were also consistent with the particle size obtained for CV-E, in which particle size for CV-E (4 °C) remained stable with no aggregation due to the high electrostatic repulsion. Meanwhile, for CV-E (25 °C and 45 °C), zeta potential decreased rapidly on day 7 and day 1, respectively, which is consistent with the particle aggregation that also occurred on day 7 for CV-E (25 °C) and day 1 for CV-E (45 °C).

CV-E was the most unstable, especially at 25 °C and 45 °C, as particle size aggregation occurred rapidly alongside a decrease in zeta potential. This is also consistent with the particle size results, in which particle aggregation was significantly greater in CV-E (25 °C and 45 °C). Generally, MC-FD at all temperatures and CV-E (4 °C) displayed relatively favourable zeta potential during most of the storage time with a zeta potential of more than ± 20 mV. There was a slight difference in the zeta potential values of MC-FD and CV-E, possibly due to the presence of different charge-containing moieties in oil mixtures and bioactive substances [116]. Researchers have reported different results for zeta potential over storage. A study by Misni et al. [117] observed a reduction in zeta potential over time for the microencapsulation of essential oil. Santos et al. [118] and Zhu et al. [119] reported an increase and decrease in zeta potential over storage. Even though zeta potential can be used to measure the physical stability of the particles, various factors can affect the measured zeta potential value. This is because zeta potential is complex, especially for small and charged particles. When non-ionic surfactants were used, they could affect the changes in the thickness of a diffuse layer and hence the measured zeta potential [120].

### 2.11. Fatty Acid Composition Changes of Lipid Particles

**Figure 10:**
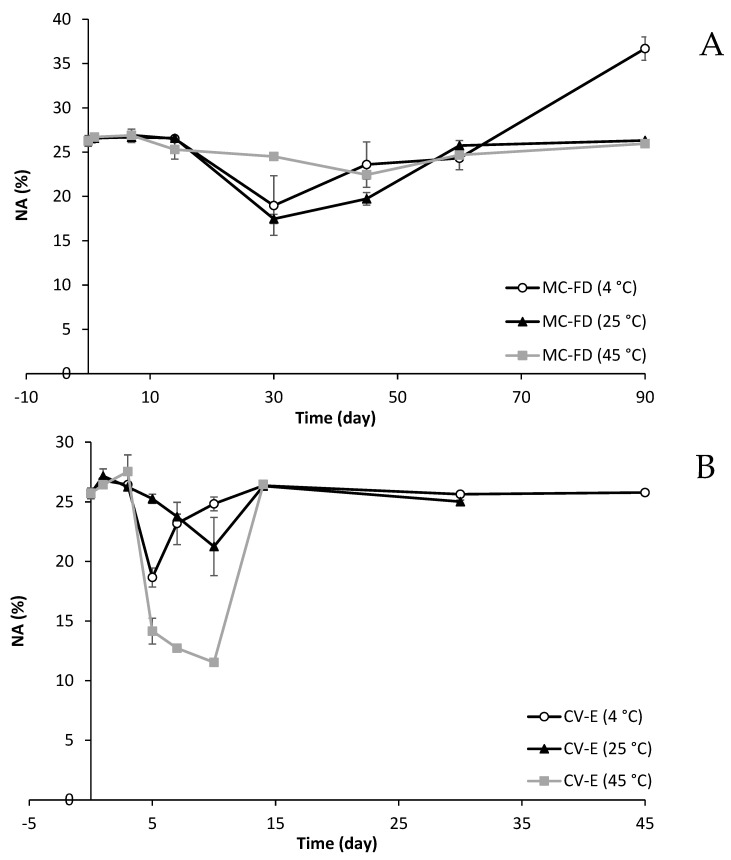

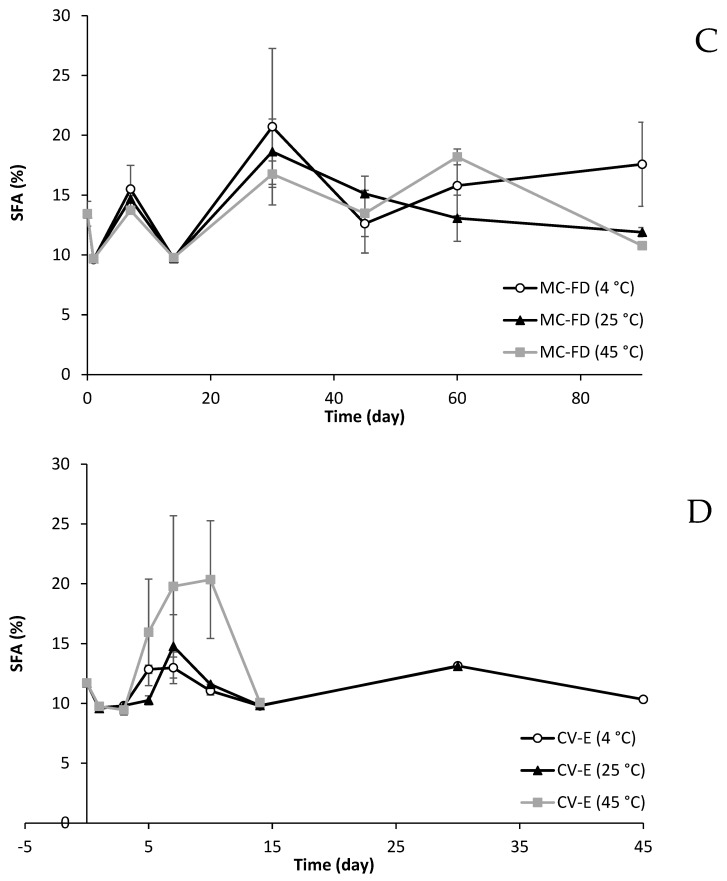

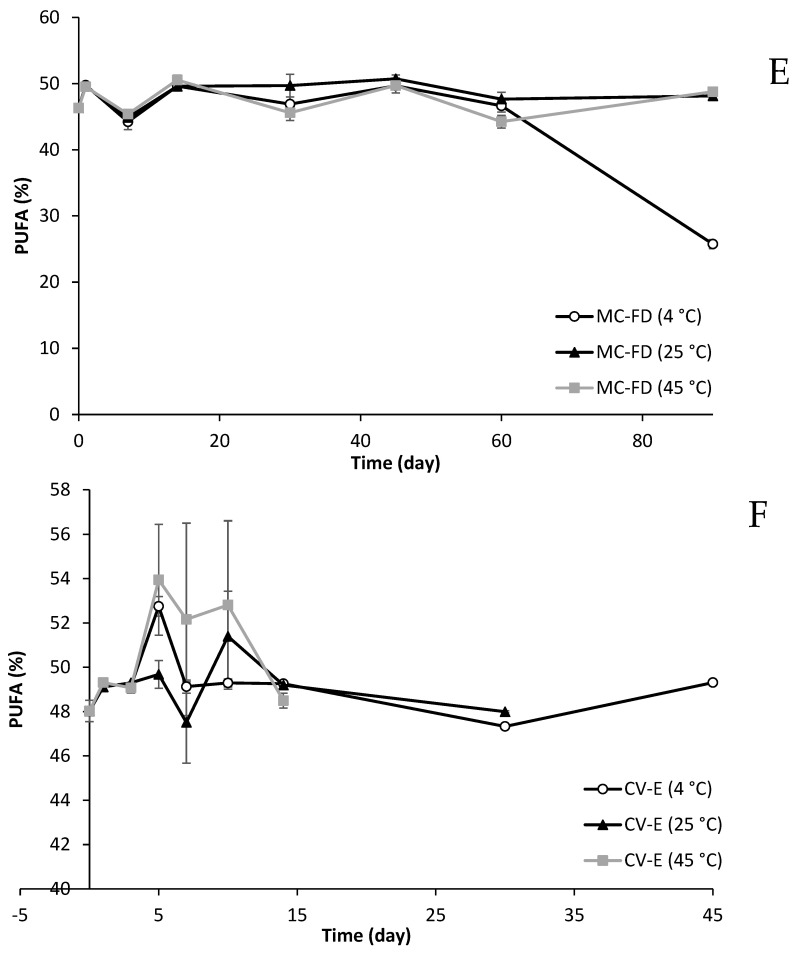
% of Nervonic acid (**A**,**B**), saturated fatty acid (**C**,**D**), and polyunsaturated fatty acid (**E**,**F**) changes of MC-FD (**A**,**C**,**E**) and CV-E (**B**,**D**,**F**) during storage at 4 °C, 25 °C, and 45 °C for up to 90 days. Large standard deviations reflect particle instability, caused by coalescence and/or aggregation.

Samples were stored at 4 °C, 25 °C, and 45 °C for up to 90 days to determine the effect of time and temperature on the fatty acid composition changes of lipid particles. Figure 10 shows the fatty acid changes in NA, SFA, and PUFA of MC-FD and CV-E during storage.

MC-FD displayed no significant changes in NA%, with a 17–26% NA, except for MC-FD (4 °C), in which NA% increased to 36.7% on day 90. This is likely due to a significant decrease in the PUFA of MC-FD (4 °C) on day 90, decreasing from approximately 47% to 25% of PUFA. This is unusual as the PUFA of MC-FD (25 °C and 45 °C) was incredibly stable throughout storage, with approximately 47% of PUFA. A likely explanation could be that MC-FD (4 °C) samples may have had leakage of oil or lipid on day 90 due to the highly porous surface of MC-FD, resulting in high oxidation of PUFA. The other MC-FD samples at 25 °C and 45 °C may have managed to maintain protection against oil leakage. The SFA of MC-FD fluctuates throughout storage, ranging from 10 to 20%. Based on the results, MC-FD displayed great stability, with no significant changes in the fatty acid composition except for day 90 MC-FD (4 °C), indicating that it has superb chemical stability even at a higher temperature.

CV-E (4 °C and 25 °C) was relatively stable, with some fluctuations between 18 and 27% NA during storage. However, CV-E (45 °C), with an initial NA% of 25%, started decreasing to 14%, respectively, on day 5 and subsequently increased to 26% on day 14. The decrease in NA% is in agreement with an increase in SFA between day 5 and day 10, and after day 14, SFA% remained similar to the initial obtained SFA%. PUFA for CV-E (4 °C) remained incredibly stable at approximately 48% during storage. CV-E (25 °C and 45 °C) displayed high physical instability, with coalescence and Ostwald ripening occurring, as observed in the particle size and the visual observation, which is the reason why there are large variations in SFA%, PUFA%, and NA% affected by lipid oxidation. A reasonable explanation could be the non-homogeneity of samples during analysis, as visual observation shows phase separation and creaming occurring as early as day 5 during storage, even though all samples were mixed before analysis to ensure homogeneity.

MC-FD displayed superior stability as there were minimal changes in NA, SFA, and PUFA. CV-E was very unstable as there were a lot of fluctuations, especially for samples at a higher temperature, and coalescence was observed visually. CV-E (25 °C and 45 °C) samples could not be analysed after day 30 and day 14, respectively, as they became solid. However, CV-E (4 °C) demonstrated superb stability as there were minimal fatty acid changes. It has been reported that fatty acids such as linoleic acid and linolenic acid esterified to PL may have enhanced protection against oxidation [121].

## 3. Materials and Methods

### 3.1. Materials

The NA-enriched SPC was prepared from lipase-catalysed acidolysis of phosphatidylcholine and NA [4]. The SPC consisted of 46.72% nervonic acid (C24:1), 31.8% linoleic acid (C18:2), 6.72% linolenic acid (C18:3), 6.12% oleic acid (C18:1), 4.41% palmitic acid (C16:0), 2.52% lignoceric acid (C24:0), 1.09% erucic acid (C22:1), and 0.61% stearic acid. Whey protein isolate (WPI) was supplied by Fonterra Co-operative Group Limited (Auckland, New Zealand), and maltodextrin (MD), sodium caseinate, gum arabic (GA), and OSA starch (OSA) were purchased from Ingredion (Auckland, New Zealand). Pepsin from porcine gastric mucosa (250 units/mg), porcine lipase (100–400 units/mg), and bile extract were purchased from Sigma-Aldrich (Castle Hill, Australia). The chemicals used include iron (III) chloride anhydrous from ECP Ltd. (Auckland, New Zealand), iron (II) sulphate heptahydrate from Sigma-Aldrich (Castle Hill, Australia), ammonium thiocyanate from Sigma-Aldrich (Castle Hill, Australia), barium chloride from ECP Ltd. (Auckland, New Zealand), 1,1,3,3-tetraethoxypropane from Sigma-Aldrich (Castle Hill, Australia), and thiobarbituric acid (TBA) purchased from Sigma-Aldrich (Castle Hill, Australia). All chemical reagents used were of analytical grade. Double-distilled water from a water purification system was used to prepare all solutions.

### 3.2. Emulsion and Microcapsule Preparation

The procedure of emulsion preparation was conducted according to the method of Chen et al. [23] with modifications. The pre-emulsions were prepared by adding the aqueous phase (emulsifier and Milli-Q water) dropwise to the lipid phase (oil and SPC) to obtain the pre-emulsions with a high shear homogeniser, IKA T18 basic Ultra-Turrax (IKA-Werke GmbH & Co. KG, Staufen im Breisgau, Germany) at 13,000 rpm for 1 min. The prepared formulations were sonicated with an ultrasonic probe homogeniser, Sonic Ruptor 250 (Omni International, Newtown, CT, USA), with power at an amplitude of 60% with 70% pulser for 2 min. The emulsion was left to cool at room temperature and pre-frozen at −80 °C for 12 h before lyophilisation at −80 °C for 24 h with a freeze-dryer (FreeZone 12 Plus, Labconco, MO, USA). The obtained MC-FD was stored at 25 °C for further analysis.

The CV-E was produced with 30% (*w*/*w*) of solid content (wall and core material) and 70% (*w*/*w*) of the aqueous phase. The core material contained 40% soybean oil and 60% SPC. The wall materials/emulsifiers, including whey protein isolate (WPI), maltodextrin (MD), gum arabic (GA), and OSA starch, were screened to select the formulation with the lowest particle size, PDI, zeta potential, and EE. The formulation of the core:wall ratio (1:1 to 1:4) was optimised for MC-FD preparation.

### 3.3. Determination of Particle Size, Polydispersity (PDI), Zeta Potential, and Entrapment Efficiency

The average particle size, PDI, and zeta potential of CV-E and MC-FD were measured by dynamic light scattering (DLS) (Zetasizer Nano ZS, Malvern Instruments Ltd., Worcestershire, UK), at a temperature of 25 °C (pH 7.0). The samples were diluted ten-fold with Milli-Q water to reduce the opalescence before the measurements. Data analysis was performed using the DTS software version 6.x.

For the in vitro digestion samples, a static light scattering device (Mastersizer 2000, Malvern Instruments Ltd., Worcestershire, UK) was used to measure particle size distribution, and the DLS (Zetasizer Nano ZS) was used to measure zeta potential. Initial and small intestine samples were diluted by 3-(N-morpholino)propanesulfonic acid (MOPS) buffer (pH 6.9), and the stomach samples were diluted with acidified water (pH 1.5) to avoid multiple scattering effects. The average particle sizes are reported as the surface-weighted mean diameter (d_3,2_).

The EE of the CV-E and MC-FD was measured according to the method by Hogan et al. [122] with modification. The emulsion sample (1 g) was extracted with petroleum ether (5 mL) for 5 min under constant agitation with a laboratory shaker. The upper phase (petroleum ether) containing extracted lipid was transferred into a pre-weighed round-bottom flask. The lower aqueous phase was re-extracted with 2 mL petroleum ether. The organic phase extracts were combined, and the solvent was evaporated from the sample with a rotary evaporator. The sample was dried in an oven at 103 °C for 1 h and left to cool before weighing the flask again. The EE was calculated using the following equation.

$$EE\%=\frac{Total\;SPC-Free\;SPC}{Total\;SPC}\times 100$$

Total oil is the amount of oil added to the formulation.

### 3.4. Fatty Acid Composition

The fatty acid composition was determined according to Wei et al. [123]. An internal standard methyl heptadecanoate (C17:0) was added. Analysis of FAMEs was carried out on gas chromatography with a flame ionisation detector (GC-FID) (Agilent 6890N, Palo Alto, CA, USA). FAMEs were identified based on the retention times of standards. The concentration of FAMEs was determined from the calibration curves of the measured peak area ratios.

### 3.5. Differential Scanning Calorimetry (DSC)

The melting point and crystallisation behaviour of the samples were determined with a differential scanning calorimeter DSC-7 (Perkin Elmer, Norwalk, CT, USA). Approximately 5 mg of each sample was placed in aluminium pans and hermetically sealed. The samples were heated from 25 to 300 °C with a heating rate of 10 °C/min under nitrogen purge and with an empty aluminium pan as a reference standard.

### 3.6. Fourier Transform Infrared Spectroscopy (FTIR)

At room temperature, the infrared spectra of MC-FD and the pure ingredients were measured with a Fourier Transform Infrared (FTIR) spectrometer, Spectrum 400, FT-IR/FT-FIR spectrometer (Perkin Elmer, Waltham, MA, USA). The measurements were recorded in the frequency range of 4000–400 cm^−1^.

### 3.7. Scanning Electron Microscopy (SEM)

The surface morphologies of the CV-E and MC-FD were investigated using a scanning electron microscope TM3030Plus (Hitachi, Tokyo, Japan) at different magnifications. The emulsion samples were diluted with deionised water. A few drops of the diluted emulsion were placed on stubs covered with an aluminium slab and dried at room temperature.

### 3.8. In Vitro Digestion

CV-E and MC-FD were passed through a gastrointestinal tract (GIT) model that simulates gastric and small intestine digestion. The digestion was conducted according to the methods by Zhang et al. [15], Chung et al. [124], and Kosaraju et al. [125] with slight modifications. The specific details for digestion are listed below.

Simulated gastric fluid (SGF): SGF was initially prepared by dissolving 0.1 g of NaCl in Milli-Q water (~40 mL), and the solution was adjusted to pH 1.5 using 1 M HCl. Subsequently, pepsin (0.15 g) was dissolved in the solution, and the volume was made up to 50 mL with Milli-Q water. The solution was prepared fresh immediately before each digestion experiment.

Simulated intestinal fluid (SGI): Buffer solution was prepared by dissolving 3-(N-morpholino)propanesulfonic acid (MOPS) (0.628 g) in Milli-Q water (~90 mL) and the pH was adjusted to 6.9 using 1 M HCl. The final volume was made up to 100 mL with Milli-Q water. The electrolyte solution was prepared by dissolving NaCl (0.6435 g) and CaCl_2_ (0.167 g) in MOPS buffer (100 mL). The pH of the solution was adjusted to 6.9 using 2 M NaOH. The bile salt solution was prepared by stirring approximately 2 h of bile salt (2 g) until it dissolved in 100 mL of MOPS buffer. Porcine lipase (1 g) was stirred with a magnetic stirrer for approximately 4 h at room temperature until it dissolved. The solution was prepared fresh daily.

Initial digestion: 20 mL of CV-E and MC-FD mixed with deionised water containing 2 wt.% lipids were placed in a centrifuge tube (50 mL).

Gastric digestion: 20 mL of emulsion samples was mixed with 20 mL of SGF containing 0.0032 g/mL pepsin and the pH was adjusted to 2. The solution was then incubated at 37 °C for 2 h with an agitation speed of 200 rpm in a shaking water bath to mimic stomach conditions.

Small intestine digestion: 30 g of “chyme” samples from the gastric digestion was placed into a 100 mL glass beaker in a water bath at 37 °C. A total of 1.5 mL of SIF was added to the sample solution, followed by 3.5 mL of bile salt solution with constant stirring. The pH of the sample solution was adjusted back to 7, adding 2.5 mL of lipase solution to the sample. An auto titrator (Metrohm Inc., Riverview, FL, USA) was used for constant stirring to monitor and maintain the pH at 7 by titrating 0.1 M NaOH solution into the sample solution at 37 °C for 2 h. The percentage of free fatty acids (FFAs) released was calculated from the number of moles of sodium hydroxide (NaOH) required to neutralise the FFA using the formula listed below.

$$FFA=100\times \frac{V(NaOH)\times m(NaOH)\times M(lipid)}{W(lipid)\times 2}$$

V(NaOH) = volume of sodium hydroxide to neutralise the FFAs (mL)

M(NaOH) = molarity of sodium hydroxide solution (0.1 M)

M (lipid) = molecular weight of lipid (g/mol)

W (lipid) = total weight of lipid initially present (g)

### 3.9. Confocal Scanning Laser Microscopy (CLSM)

The microstructures of all samples were observed using CLSM Olympus FV1000 confocal microscope (Olympus Corporation, Tokyo, Japan) with a 100× oil immersion objective lens. Before analysis, 2 mL samples were mixed with 0.1 mL Nile Red solution (1 mg/mL ethanol) to dye the oil phase and 0.1 mL FITC solution (10 mg/mL dimethyl sulfoxide) to dye the protein. The excitation and emission spectra for Nile Red were 543 nm and 605 nm, respectively, and for FITC were 488 nm and 515 nm, respectively. The sample aliquot was pipetted onto a microscope slide, covered by a cover slip, and the microstructure images were acquired using image analysis software (Fluoview FV10-ASW 4.2 Software, Olympus).

### 3.10. Storage Stability Tests

CV-E and MC-FD were stored at 4 °C, 25 °C, and 45 °C with 75% relative humidity in a desiccator wrapped with aluminium foil without any exposure to light. The storage stability test was evaluated based on the particle size, PDI, zeta potential, peroxide value (PV), thiobarbituric reactive substances (TBARS), and fatty acid composition (Table 5). CV-E samples were analysed on days 0, 1, 3, 5, 7, 10, 14, and 30, while the MC-FD were analysed on days 0, 1, 7, 14, 30, 45, 60, and 90.

### 3.11. Primary and Secondary Lipid Oxidation Products

A rapid spectrophotometric method by Hornero-Méndez et al. [126] was utilised for the calculation of peroxide value (PV) using the equation below.

$$PV\frac{meq}{kg}=\frac{A_{a}-A_{b}}{55.84\times 2\times m\times W_{s}}$$

A_a_ = absorbance of the sample at 470 nm

A_b_ = absorbance of the blank at 470 nm (both absorbances corrected after subtracting absorbance at 670 nm)

m = slope of the calibration curve

W_s_ = sample weight (g)

The data were reported as mean ± standard deviation of duplicate measurements.

The secondary oxidation products were evaluated by quantifying the thiobarbituric reactive substances (TBARS) according to the method by Wang et al. [127]. Thiobarbituric acid (TBA) solution was obtained by dissolving 15 g of trichloroacetic acid (15% *w*/*v*) and 0.375 g of TBA (0.375%) in 100 mL of 0.25 mol/L HCl. The samples (0.2 mL) were mixed with 1.8 mL of Milli-Q water and 4 mL of TBA solution. The sample mixtures were then boiled in a water bath for 15 min and cooled to room temperature and, lastly, the mixtures were centrifuged at 2000× *g* for 15 min. The colour intensity produced from the reaction between TBA and malondialdehyde, a crucial by-product of lipid peroxidation, was measured at 532 nm. The standard curve of 1,1,3,3-tetraethoxypropane was utilised to determine the malondialdehyde concentrations.

### 3.12. Statistical Analysis

Experiments were performed in duplicate and triplicate. The results were presented as mean ± standard deviation (SD). The statistical significance level was determined at 95% confidence limit (*p* < 0.05) and was statistically analysed by One-Way Analysis of Variance (ANOVA) with statistical software (SPSS). The Tukey and Duncan tests were applied to determine the significant differences between the means at a probability level of 5% (*p* ≤ 0.05).

## 4. Conclusions

This study involved the production and encapsulation of NA-enriched SPC in emulsion and microcapsule format using WPI and MD as wall materials. This was followed by the study of the physical and chemical properties of the resulting emulsion and microcapsules. Lastly, the potential gastrointestinal fate of the encapsulated lipid was examined and compared. The composition of the wall materials and core:wall ratio strongly influences the properties of CV-E and MC-FD. The emulsion morphology displays regular round-shaped particles but aggregated into large particles due to water removal, while MC-FD showed irregular, sharp, and broken glass-like surfaces. Based on the screening and the optimisation of the wall materials, WPI and MD at the core:wall ratio of 1:3 gave the smallest particle size with high zeta potential, EE of 99%, and the most uniform particle size distribution, as these properties promote higher stability.

During digestion, the particle size of CV-E and MC-FD increased after exposure to simulated digestion conditions, which was attributed to droplet aggregation (flocculation or coalescence) and lipid digestion (formation of micelles, vesicles, and calcium soaps). The electrical properties changed through the digestion phases, which was attributed to the digestion and displacement of protein molecules from the oil droplet surfaces and the formation of other colloidal particles, such as mixed micelles. The samples were rapidly digested and displayed superb stability during digestion. MC-FD had the highest FFA release of 79.54%, followed by CV-E with 72.1% and SPC with only 29.82%. This information obtained from the results is crucial as it can be used to control lipid absorption and the bioaccessibility of the bioactive components within the GIT.

This study investigates the physical and chemical stability of CV-E and MC-FD by evaluating the primary and secondary oxidative stability, particle size, PDI, zeta potential, visual appearance, and fatty acid composition changes during storage. Based on the results, it was observed that freeze-dried samples were generally more stable than emulsion samples. CV-E displayed very low physical stability, with a significant increase in particle sizes due to coalescence throughout storage. Therefore, MC-FD could be considered a product to supplement NA, as it has also demonstrated excellent storage stability at 4 °C. Compared to previous studies such as that by Jin et al. [22], which encapsulated NA using soy and whey proteins in nanoemulsion form, this study is novel in its use of NA-enriched phospholipid as the core material and the conversion of emulsions into freeze-dried microcapsules. Furthermore, this work uniquely investigates the oxidative and digestive stability of NA in both emulsion and powder form, offering deeper insight into the suitability of these formats for long-term storage and enhanced bioaccessibility.

## Figures and Tables

**Table 1:** Screening of wall materials to produce CV-E (core:wall ratio 1:4) by evaluating the particle size, PDI, zeta potential, and entrapment efficiency (EE).

Wall Material		Particle Size (nm)	PDI	Zeta Potential	EE%
WPI		193 ± 3 ^a^	0.259 ± 0.05 ^ab^	−35.2 ± 1.1 ^a^	99.2 ± 0.9 ^a^
WPI	GA	191 ± 1 ^a^	0.331 ± 0.01 ^b^	−23.2 ± 0.9 ^b^	99.7 ± 0.2 ^a^
WPI	MD	175 ± 6 ^a^	0.169 ± 0.02 ^a^	−32.2 ± 1.3 ^a^	99.5 ± 0.1 ^a^
WPI	OSA	228 ± 19 ^a^	0.308 ± 0.00 ^ab^	−21.3 ± 0.7 ^b^	99.6 ± 0.1 ^a^

Values are presented as mean SD ± (n = 2). Different letters indicate whether they are significantly different for each column (Tukey, *p* < 0.05).

**Table 2:** Selection of wall material’s ratio to produce conventional emulsion by evaluating the particle size, PDI, zeta potential, and entrapment efficiency (EE).

Core:Wall	Particle Size (nm)	PDI	Zeta Potential	EE%
1:1	924 ± 64 ^a^	0.777 ± 0.05 ^a^	−54.8 ± 4.9 ^a^	95.0 ± 0.2 ^a^
1:2	369 ± 9 ^b^	0.685 ± 0.12 ^a^	−58.9 ± 1.1 ^a^	97.2 ± 0.1 ^a^
1:3	188 ± 11 ^c^	0.177 ± 0.02 ^b^	−33.0 ± 1.6 ^b^	99.5 ± 0.3 ^b^
1:4	171 ± 7 ^c^	0.176 ± 0.02 ^b^	−31.5 ± 3 ^b^	99.5 ± 0.1 ^b^

Values are presented as mean SD ± (n = 3). Different letters indicate whether they are significantly different for each row (Tukey, *p* < 0.05).

**Table 3:** Melting point and enthalpy of conventional emulsion and freeze-dried microcapsule.

		Melting Point (°C)	Enthalpy (J/g)
MC-FD	1st peak	151.63	5.997
	2nd peak	161.77	105.3
	3rd peak	268.42	24.16
CV-E		105.99	1622

**Table 4:** Fatty acid composition of CV-E, MC-FD, and SPC before and after digestion.

Fatty Acid	Before Digestion (CV-E)	After Digestion (CV-E)	Before Digestion (MC-FD)	After Digestion (MC-FD)	Before Digestion (SPC)	After Digestion (SPC)
C16:0	7.08 ± 0.11 ^c^	3.8 ± 0.64 ^ab^	8.13 ± 0.69 ^c^	6.04 ± 0.07 ^bc^	1.91 ± 0.23 ^a^	3.16 ± 0.01 ^a^
C18:0	2.95 ± 0.09 ^b^	1.71 ± 0.33 ^a^	3.58 ± 0.38 ^b^	2.96 ± 0.03 ^b^	0.54 ± 0.09 ^a^	1.34 ± 0.07 ^a^
C18:1	14.55 ± 0.42 ^d^	10.59 ± 0.15 ^c^	13.96 ± 0.08 ^d^	10.22 ± 0.04 ^c^	5.03 ± 0.19 ^b^	4.24 ± 0.03 ^a^
C18:2	43.47 ± 0.42 ^b^	32.81 ± 0.81 ^b^	41.78 ± 0.3 ^b^	32.73 ± 0.08 ^b^	34.88 ± 1.39 ^b^	26.74 ± 0.27 ^a^
C18:3	4.56 ± 0.06 ^c^	3.17 ± 0.07 ^b^	4.54 ± 0.04 ^c^	3.19 ± 0.01 ^b^	3.58 ± 0.18 ^b^	2.61 ± 0.08 ^a^
C20:0	0.74 ± 0.00 ^a^	0.99 ± 0.03 ^b^	0.77 ± 0.02 ^a^	0.95 ± 0.01 ^b^	1.21 ± 0.01 ^c^	1.24 ± 0.03 ^c^
C24:0	0.94 ± 0.01 ^a^	1.54 ± 0.00 ^b^	0.96 ± 0.02 ^a^	1.53 ± 0.01 ^b^	2.7 ± 0.23 ^c^	1.96 ± 0.06 ^b^
C24:1	25.72 ± 0.45 ^a^	45.39 ± 0.04 ^b^	26.28 ± 0.61 ^a^	42.39 ± 0.04 ^b^	50.16 ± 1.86 ^c^	58.72 ± 0.09 ^d^
Total SFA	11.71	8.04	13.44	11.48	6.36	7.7
Total USFA	88.3	91.96	86.56	88.53	93.65	92.31
Total MUFA	40.27	55.98	40.24	42.61	55.19	62.96
Total PUFA	48.03	35.98	46.32	35.92	38.46	29.35

Values are presented as mean SD ± (n = 2). Different letters indicate whether they are significantly different for each row (Tukey, *p* < 0.05). Saturated fatty acid (SFA), unsaturated fatty acid (UFA), monounsaturated fatty acid (MUFA), and polyunsaturated fatty acid (PUFA).

**Table 5:** Storage stability test conditions, sampling points, and analysis of encapsulated samples.

Samples	CV-E	MC-FD
Storage conditions	4 °C/25 °C/45 °C, 75% (relative humidity)	
Frequency (days)	1, 3, 5, 7, 10, 14, 30	1, 7, 14, 30, 45, 60, 75, 90
Analysis	Particle size, PDI, zeta potential, PV, TBA test, and fatty acid content	

## Data Availability

The data presented in this study are available on request from the corresponding author.

## Supplementary Materials

The following supporting information can be downloaded at: https://www.mdpi.com/article/10.3390/molecules30092007/s1, Figure S1: Particle size distributions as a function of the intensity % obtained from a dynamic light scattering of the CV-E with a combination of different wall materials (A) and CV-E (WPI:MD) with core:wall ratio of 1:1–1:4 (B).
