# Supplementary material for: Preparation, Digestion, and Storage of Microencapsulated Nervonic Acid-Enriched Structured Phosphatidylcholine
Source: Molecules. 2025 Apr 30;30(9):2007. doi: 10.3390/molecules30092007 (PMC12073651; doi:10.3390/molecules30092007)
Supplement: Supplementary file 1 [file molecules-30-02007-s001.zip › molecules-3588412-supplementary.pdf]

Supplementary Materials

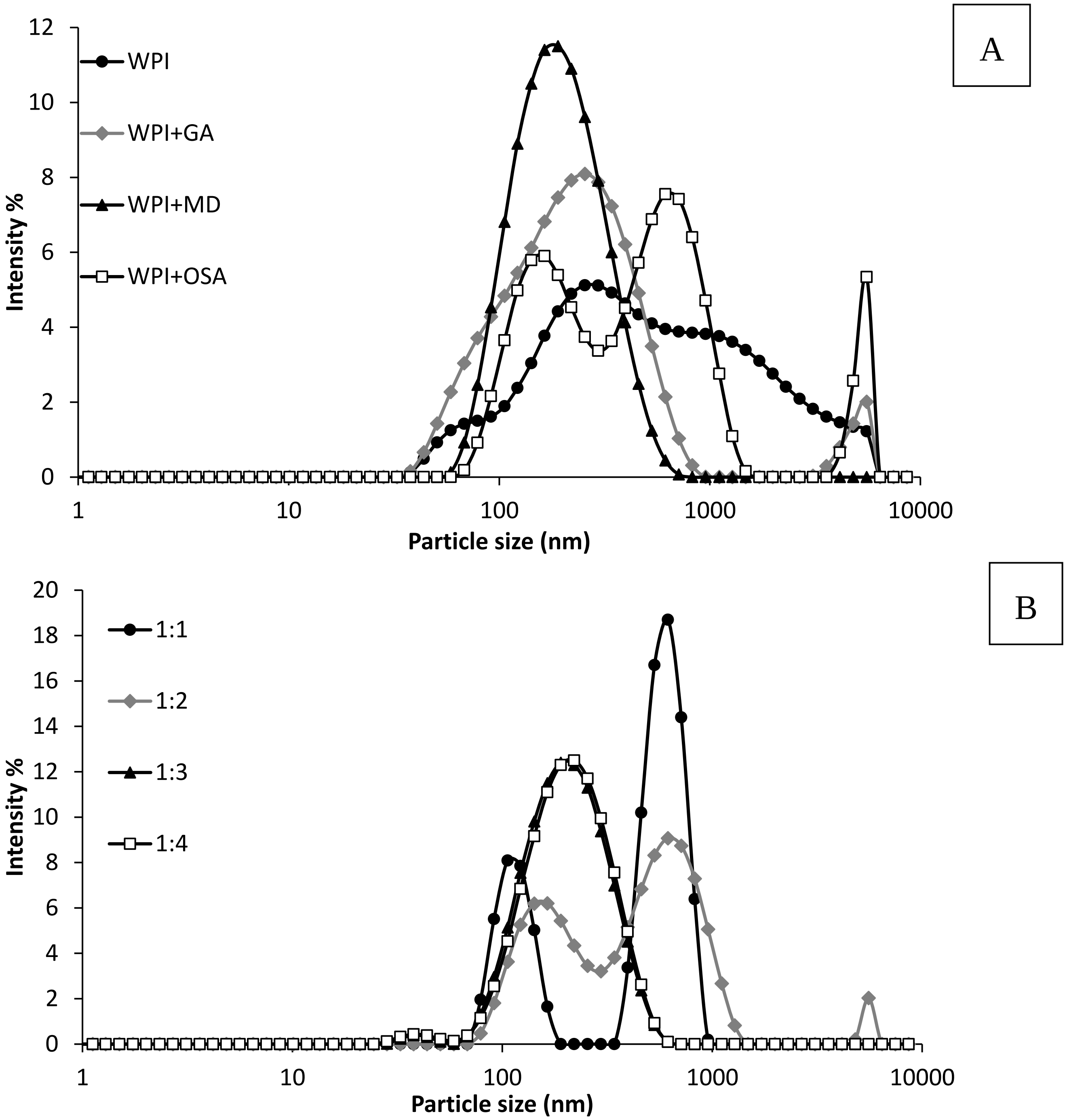

Supplementary Figure 1: Particle size distributions as a function of the intensity % obtained from a dynamic light scattering of the CV-E with a combination of different wall materials (A) and CV-E (WPI:MD) with core:wall ratio of 1:1-1:4 (B).
